# Contextual boundaries of personal resource translation: dual-path associations of role overload and job autonomy with job satisfaction among sports practitioners

**DOI:** 10.3389/fpsyg.2026.1844634

**Published:** 2026-10-07

**Authors:** Li Zhang, Jinjin Ren, Ke Sun

**Affiliations:** 1 Pingdingshan Polytechnic College, Pingdingshan, China; 2 Keimyung College University (KMCU), Daegu, Republic of Korea; 3 Pingdingshan University, Pingdingshan, China

**Keywords:** contextual boundary of personal resource translation, job autonomy, job satisfaction, job self-efficacy, role overload, sports practitioners, work engagement

## Abstract

**Background:**

Sports practitioners work under sustained job demands while also relying on varying degrees of control over how work is organized. Whether personal resources are associated with occupational experience in the same way across different work structures remains uncertain. This study examined the dual-path associations of role overload and job autonomy with job satisfaction through work engagement and assessed the conditional role of job self-efficacy.

**Methods:**

A *post hoc*, theory-guided analysis was conducted using data from a broader cross-sectional survey of 358 sports practitioners in China. Confirmatory factor analysis evaluated the measurement model, and regression-based conditional-process analyses were conducted with adjustment for sex.

**Results:**

The five-factor measurement model showed a good fit (Comparative Fit Index [CFI] = 0.952, Tucker–Lewis Index [TLI] = 0.944, Root Mean Square Error of Approximation [RMSEA] = 0.051, Standardized Root Mean Square Residual [SRMR] = 0.058). Role overload was negatively associated with job satisfaction (B = −0.355, *p* < 0.001), whereas job autonomy was positively associated with job satisfaction (B = 0.293, *p* < 0.001). Both pathways showed indirect associations through work engagement. Job self-efficacy showed an asymmetric conditional pattern. The role overload × job self-efficacy interaction was negative (B = −0.152, *p* = 0.008), and the index of moderated mediation was −0.063, 95% bootstrap CI [−0.111, −0.016]. This negative interaction remained in the joint model that simultaneously included role overload and job autonomy (B = −0.124, *p* = 0.038). Johnson–Neyman analysis indicated that the adjusted association between role overload and work engagement was significantly negative when job self-efficacy was above 3.36. In contrast, job autonomy retained stable positive main and indirect associations, whereas its interaction with job self-efficacy was not robustly supported.

**Conclusion:**

The asymmetric conditional pattern suggests that the association of job self-efficacy with work engagement may vary across demand-intensive and autonomy-related work conditions. This context-dependent interpretation provides a focused proposition for longitudinal and work-design research.

## 1 Introduction

Work in sport is characterized by sustained demands. Coaches, athletes, referees, and technical-support personnel must manage training and competition, performance evaluation, organizational coordination, role communication, and time pressure; in high-performance settings, frequent travel, job insecurity, and public scrutiny can add further strain. Research in competitive and high-performance sport indicates that occupational experience is shaped not by a single stressor but by the joint configuration of organizational demands and resources, including workload, organizational structure, interpersonal coordination, and access to resources (Arnold and Fletcher, 2012; Arnold et al., 2019; Simpson et al., 2024; Mercer et al., 2025). The sport-work context therefore provides a useful setting in which to examine associations among specific job demands, job resources, personal resources, and occupational psychological states. In this study, role overload is used in a deliberately narrow sense: it is operationalized through quantitative workload and time–pressure content rather than the broader domain of role stress. Job autonomy captures discretion over work scheduling, professional judgment, and task execution. These dimensions are not opposite ends of a single continuum: high quantitative overload can coexist with high autonomy, and lower overload can coexist with low autonomy. Understanding sport work therefore requires separate consideration of demand intensity and action control. Classic demand-control research and meta-analytic evidence identify both dimensions as relevant to work stress and job satisfaction (Karasek, 1979; Spector, 1986). The Job Demands-Resources (JD-R) model places these relationships within a broader framework of occupational psychology. Job demands require sustained physical, cognitive, or emotional expenditure, whereas job resources support goal attainment, motivation, and professional growth (Demerouti et al., 2001; Bakker and Demerouti, 2007, 2017). Longitudinal meta-analysis suggests that demands and resources show relatively stable, but not simply symmetric, associations with subsequent occupational states (Lesener et al., 2019). A meta-analysis of 113 samples further showed consistent associations of job and personal resources with work engagement, with a particularly strong link between work engagement and job satisfaction (Mazzetti et al., 2023). Work engagement is therefore a plausible psychological correlate linking concrete work characteristics with global work attitudes.

An unresolved issue concerns the conditions under which personal resources may be associated with occupational outcomes. Job self-efficacy is not a generic personality advantage; it is a capability judgment about whether one can perform work tasks, organize action, and deal with work demands. Social Cognitive Theory proposes that efficacy beliefs shape action choice, effort, and persistence (Bandura, 1997). Riggs et al. (1994) demonstrated that job-related efficacy beliefs can be measured coherently in work settings and are systematically related to work attitudes and performance evaluations. Both JD–R theory and Conservation of Resources Theory provide a basis for considering whether the association of such capability beliefs with work outcomes varies across work conditions. JD–R research commonly treats personal resources as conditions that support engagement, yet personal resources do not consistently buffer all job demands (Xanthopoulou et al., 2007). Conservation of Resources Theory likewise emphasizes that resource investment is sustainable when it protects existing resources or produces resource gains (Hobfoll et al., 2018). These perspectives suggest that the occupational relevance of job self-efficacy may vary with the surrounding pattern of demands and control. We use the contextual boundary of personal resource translation as a theoretical label for this proposed context dependence.

Against this background, the present study places role overload and job autonomy within one dual-path framework, uses work engagement as an indirect association between work characteristics and job satisfaction, and examines whether job self-efficacy changes the first-stage association in each path. The framework distinguishes quantitative demand intensity, represented by the present role-overload indicator; action control, represented by job autonomy; and capability belief, represented by job self-efficacy. Comparing conditional patterns across the two paths allows an empirical assessment of whether the same capability belief shows different associations across contrasting work conditions. Ability-control mismatch provides one theoretical lens for interpreting the role-overload pathway.

## 2 Theoretical background and hypotheses

### 2.1 Dual pathways within the JD–R framework

The JD–R model does not require a fixed list of stressors; instead, it classifies work characteristics by function. Job demands are aspects of work that require sustained physical, cognitive, or emotional effort, whereas job resources facilitate goal attainment, motivation, and growth (Demerouti et al., 2001; Bakker and Demerouti, 2017). Sport occupations are well suited to this perspective. Elite-sport support teams must manage organizational structures, logistics, interpersonal coordination, and performance development (Arnold et al., 2019), while systematic reviews show that the consequences of stressors depend on appraisal, coping latitude, and available resources (Simpson et al., 2024; Mercer et al., 2025). Identifying specific work characteristics is therefore more informative than relying on a broad label of occupational stress.

Role overload and job autonomy serve as the starting points for the two pathways. Role overload can encompass excessive role demands relative to available time and capacity. The present six-item operationalization is narrower: it captures the quantitative core of overload through work volume, pace, effort, time pressure, rushing, and difficulty keeping up. This content overlaps established role-overload formulations emphasizing excessive work and insufficient time (Bolino and Turnley, 2005) and the pace/volume content of the Quantitative Workload Inventory (Spector and Jex, 1998). Throughout this study, ‘role overload’ therefore refers specifically to this quantitative workload/time–pressure facet, not to broader role stress, role ambiguity, or role conflict (Kahn et al., 1964; Rizzo et al., 1970). Job autonomy reflects discretion over task order, professional methods, and task execution and therefore represents action control. Together, the two constructs capture distinct work-design dimensions: quantitative demand intensity and control.

JD–R theory predicts that hindrance demands are more likely to coincide with resource depletion and reduced motivation, whereas job resources are more likely to support engagement and goal attainment (Crawford et al., 2010; Bakker et al., 2014). Longitudinal evidence is broadly consistent with this pattern, showing stable associations of demands and resources with subsequent burnout, engagement, and related occupational outcomes (Hakanen et al., 2008; Lesener et al., 2019). Research among sport coaches likewise identifies job resources as closely related to engagement and sustained demands as related to poorer occupational states (Kaski and Kinnunen, 2021). We therefore position work engagement between the two work-characteristic pathways and examine whether the demand and resource pathways show different conditional patterns.

### 2.2 Role overload, job autonomy, and job satisfaction

#### 2.2.1 Role overload and job satisfaction

Role overload, in the quantitative sense examined here, arises when the amount or pace of work exceeds what can reasonably be managed within available time and capacity. Established role-overload measures commonly emphasize excessive work volume and insufficient time (Bolino and Turnley, 2005), while quantitative workload measures similarly focus on work pace and amount (Spector and Jex, 1998). In sport work, training, competition, administrative duties, and *ad hoc* coordination can converge within restricted time windows. Conservation of Resources Theory provides one basis for expecting less favorable work evaluations under such conditions: sustained high quantitative demands require continued expenditure of time, attention, and energy (Hobfoll, 1989; Halbesleben et al., 2014; Hobfoll et al., 2018). Cross-occupational evidence links role overload with lower engagement and poorer work outcomes (Zhang et al., 2022; Guo et al., 2025), and research among sport coaches similarly associates higher job demands with poorer psychological well-being (Potts et al., 2023).

*H1a*: Role overload is negatively associated with job satisfaction among sports practitioners.

#### 2.2.2 Job autonomy and job satisfaction

Job autonomy reflects the degree of actual control over the work process. For sports practitioners, such control may involve training arrangements, task sequencing, professional methods, and on-site decisions. The value of autonomy lies not simply in having choices but in being able to adapt work based on professional judgment and situational feedback rather than following predetermined procedures. Classic work-control research and meta-analytic evidence link greater decision latitude with higher job satisfaction and motivation and with lower role stress (Karasek, 1979; Spector, 1986). Self-Determination Theory provides a complementary explanation: autonomy support facilitates basic psychological need satisfaction and more self-determined forms of motivation. A recent meta-analysis of 192 workplace studies similarly showed systematic associations among need support, need satisfaction, autonomous motivation, and positive work outcomes (Hagger and McAnally Star, 2026). Together with prospective evidence on job autonomy and psychological well-being (Clausen et al., 2022), these findings support the following hypothesis.

*H1b*: Job autonomy is positively associated with job satisfaction among sports practitioners.

### 2.3 Indirect associations through work engagement

Job satisfaction reflects an individual’s overall evaluation of work and the work environment, whereas work engagement describes vigor, dedication, and absorption during work (Kahn, 1990; Schaufeli et al., 2002). The constructs occupy different psychological levels but are consistently related. In a JD–R meta-analysis of 113 samples, Mazzetti et al. (2023) found stable associations of job and personal resources with work engagement and a particularly strong link between work engagement and job satisfaction; a meta-analysis among healthcare workers likewise reported a moderate-to-strong overall association between the two outcomes (Yildiz and Yildiz, 2022). In the role-overload pathway, demands that persistently exceed manageable capacity are associated with greater expenditure of attention and energy. Conservation of Resources Theory proposes that when resource loss outpaces replenishment, individuals may reduce discretionary investment to conserve remaining resources (Hobfoll, 1989, 2002). Empirical studies repeatedly link role overload with lower work engagement (Zhang et al., 2022; Guo et al., 2025), and lower engagement is in turn associated with lower job satisfaction (Orgambídez and Extremera, 2020). In the job-autonomy pathway, decision latitude allows workers to reorganize tasks, apply professional judgment, and regulate work pace in response to feedback. Autonomy can therefore strengthen the connection between work goals and personal action, making active engagement more feasible. Prior research similarly identifies work engagement as an important psychological link between autonomy and positive work outcomes (Christian et al., 2011; Rich et al., 2010; Svensson et al., 2021).

*H2a*: Role overload shows a negative indirect association with job satisfaction through work engagement; higher role overload is associated with lower work engagement, which in turn is associated with lower job satisfaction.

*H2b*: Job autonomy shows a positive indirect association with job satisfaction through work engagement; higher job autonomy is associated with higher work engagement, which in turn is associated with higher job satisfaction.

### 2.4 Job self-efficacy and contextual boundaries of personal resource translation

Job self-efficacy refers to judgments about whether one can perform specific work tasks, use existing capabilities, and deal with occupational demands. Compared with generalized self-efficacy, it is more closely tied to work behavior and work outcomes. Riggs et al. (1994) developed and validated job-related self-efficacy measures and showed systematic associations with job satisfaction, organizational commitment, and work performance. Social Cognitive Theory further proposes that efficacy beliefs influence action initiation, effort, and persistence, while opportunities and constraints in the environment shape whether those actions can produce effective outcomes (Bandura, 1997). Job self-efficacy is therefore a personal resource whose functioning is embedded in the work context. Research on personal resources within JD–R theory is consistent with this view. Xanthopoulou et al. (2007) found that self-efficacy, self-esteem, and optimism were associated with perceived job resources and work engagement but did not uniformly buffer the relationship between job demands and exhaustion. The Triple-Match Principle similarly proposes that functional correspondence among stressors, resources, and outcomes influences whether a resource becomes useful at a critical point (de Jonge and Dormann, 2006). In the present framework, job self-efficacy represents an action-oriented capability belief, whereas role overload and job autonomy represent work conditions that may constrain or expand the space within which capability can be translated into action. This functional distinction does not require the two interaction patterns to be symmetric.

#### 2.4.1 Constrained resource translation under role overload

Role overload provides an informative context for examining a potential boundary on personal-resource functioning. Individuals with higher job self-efficacy are generally more willing to act on problems and sustain effort when difficulties arise. When tasks are sufficiently controllable, persistence can improve the probability of successful problem solving. Under role overload, however, task volume, time pressure, and responsibility may approach or exceed what an individual can regulate. Additional effort cannot by itself create more time, staffing, or organizational authority, creating potential tension between capability beliefs and actual latitude for adjustment. Conservation of Resources Theory helps explain why this tension may be reflected in work engagement. Resource investment is sustainable when it preserves existing resources or generates new gains (Halbesleben et al., 2014; Hobfoll et al., 2018). When continued effort can change the task environment, job self-efficacy may support problem solving and resource gain. When effort mainly increases personal expenditure without changing the source of overload, stronger capability beliefs may sustain effort for longer in a low-conversion environment, increasing resource costs. The negative association between role overload and work engagement may therefore be stronger at higher levels of job self-efficacy. The broader proposition is that the occupational relevance of a personal resource may depend on whether the surrounding work structure permits that resource to be enacted effectively.

*H3a*: Job self-efficacy moderates the association between role overload and work engagement, such that the negative association is stronger at higher than at lower levels of job self-efficacy.

#### 2.4.2 A resource-translation channel under job autonomy

Job autonomy represents a different work structure. It gives individuals room to adjust work methods, scheduling, and task execution, allowing capability beliefs to be enacted through real decisions. Work-design research identifies autonomy as a core motivational characteristic and links method autonomy, decision autonomy, and scheduling autonomy with favorable work attitudes (Morgeson and Humphrey, 2006). If job self-efficacy provides the belief that one can perform the task and job autonomy provides latitude over how the task is performed, the two resources may be functionally complementary. Resource-gain research has described mutually reinforcing relations among efficacy beliefs, job resources, and work engagement (Llorens et al., 2007; Xanthopoulou et al., 2009; Salanova et al., 2011). Yet complementarity does not imply that additional resources will always yield stronger effects. When job autonomy already provides substantial decision and adjustment latitude, whether higher job self-efficacy produces detectable incremental gain remains an empirical question. The autonomy pathway therefore tests possible resource complementarity while providing a necessary theoretical comparison with the role-overload pathway.

*H3b*: Job self-efficacy moderates the association between job autonomy and work engagement, such that the positive association is stronger at higher than at lower levels of job self-efficacy.

#### 2.4.3 Conditional indirect associations

If job self-efficacy changes the first-stage association of role overload or job autonomy with work engagement, the indirect association of each work characteristic with job satisfaction through work engagement should also vary across levels of job self-efficacy. Following the arguments above, the role-overload pathway is expected to show a stronger negative indirect association at higher job self-efficacy, whereas the job-autonomy pathway is expected to show a stronger positive indirect association.

*H4a*: The negative indirect association of role overload with job satisfaction through work engagement is moderated by job self-efficacy and is stronger at higher levels of job self-efficacy.

*H4b*: The positive indirect association of job autonomy with job satisfaction through work engagement is moderated by job self-efficacy and is stronger at higher levels of job self-efficacy.

The dual-path theoretical structure represented by these hypotheses is shown in Figure 1.

**Figure 1:**
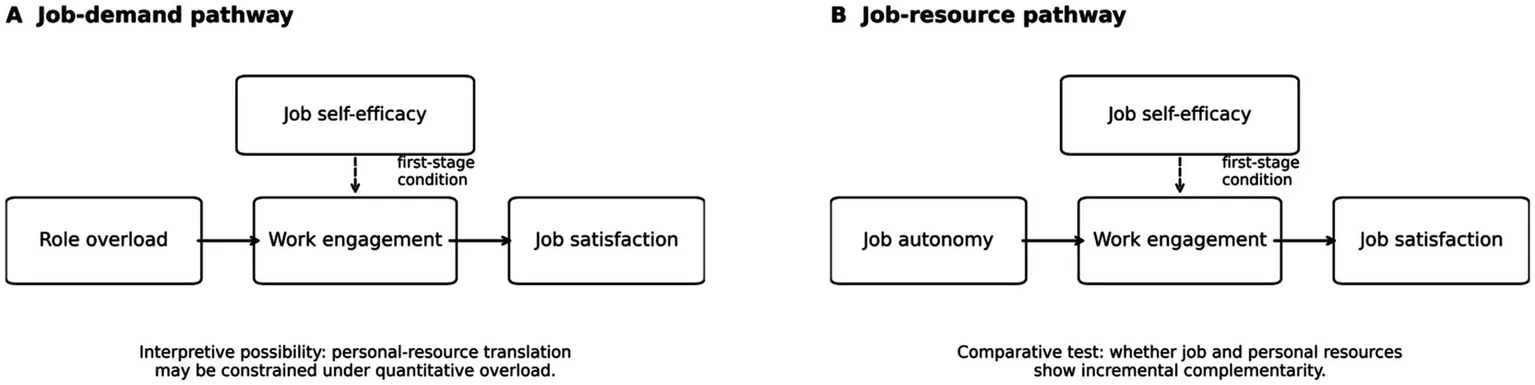
Dual-path theoretical framework for contextual boundaries of personal resource translation. ***Note.*** The figure summarizes the theory-guided conditional framework underlying H2–H4. Job self-efficacy is specified as the first-stage conditional variable in both pathways.

## 3 Methods

### 3.1 Sample and procedure

This cross-sectional, non-interventional survey was conducted in mainland China from 1 August to 30 September 2025 using paper-and-pencil questionnaires. Participants were recruited through sports bureaus, sports teams and training bases, sports associations and clubs, and acquaintance referral/snowball sampling. The recruitment frame targeted adults currently engaged in sport-related roles. Adult eligibility was verified on site before questionnaire administration by inspection of each participant’s government-issued identity document; no identity document number or other identifying information was recorded. Participants then provided written informed consent. Age was not recorded as a numeric analytic variable, and education, employment status, and occupational tenure were not collected. A total of 358 complete questionnaires were returned; the focal study variables had no missing data, and all 358 cases were included in the analyses. Because recruitment occurred through multiple offline channels and the total number of questionnaires initially distributed was not retained in a unified, verifiable record, a conventional response rate could not be calculated. No financial compensation or gifts were provided, and the data were de-identified for analysis. The study was approved by the Ethics Committee of Yancheng Institute of Technology (YIT-TY-IRB-2025-07-007; 30 June 2025). The analytic sample comprised 128 coaches, 162 athletes, and 68 referees/technical-support personnel; 203 participants were male and 155 female (Table 1). Individual-level data were available for sex and occupational role. Primary sport and broad region were recorded on the paper questionnaire but were available only as aggregate manual tallies without row-level linkage to the analytic records; these variables were therefore not included in participant-level descriptive or outcome analyses. Occupational role was used for cross-role measurement-invariance testing and role-adjusted sensitivity analyses. Primary inferential models were adjusted for sex.

**Table 1:** Sample characteristics (*N* = 358).

Category	Level	n	%
Sex	Male	203	56.7
	Female	155	43.3
Occupational role	Coach	128	35.8
	Athlete	162	45.3
	Referee/technical-support	68	19.0

Sex was coded 1 = male and 2 = female. The occupational role comprised coach, athlete, and referee/technical-support personnel. Age was not collected as a numeric variable; education, employment status, and occupational tenure were not collected. Primary sport and region were available only as aggregate paper tallies and had no row-level linkage to the analytic records.

### 3.2 Measures

The complete administered questionnaire contained 43 non-demographic measurement items covering role overload, role ambiguity, role conflict, social support, job autonomy, self-development opportunities, feedback, job self-efficacy, work engagement, and job satisfaction. The present analysis focuses on five constructs represented by 22 items: role overload (6 items), job autonomy (4 items), job self-efficacy (3 items), work engagement (3 items), and job satisfaction (6 items). The focal framework was formulated after data collection and after preliminary inspection of descriptive statistics and correlations, measurement model and reliability results, and ordinary regression and Model 4 indirect-association results. Accordingly, the analysis is *post hoc* and theory-guided. Conditional-process analyses, including Model 7 interactions, Johnson–Neyman analyses, and moderated-mediation indices, were conducted after the five-construct framework had been specified.

The five focal constructs define the theoretical contrast examined here: role overload represents quantitative demand intensity, job autonomy represents action control, job self-efficacy represents a work-domain capability belief, work engagement represents a proximal occupational state, and job satisfaction represents the focal work-evaluation outcome. Role ambiguity, conflicting role demands, social support, feedback, and self-development opportunities were part of the broader survey but address distinct questions concerning role clarity, role conflict, and alternative job resources and were outside the focal dual-path model. The selection chronology and rationale are detailed in Supplementary Table S1B. All 22 focal items were scored in the same direction, with no reverse scoring, and construct scores were calculated as the mean of their constituent items. All items used a five-point Likert scale from 1 (strongly disagree) to 5 (strongly agree). The questionnaire was a Chinese-contextualized instrument based on established constructs and item content and was adapted in wording for Chinese sport-work settings. Because the questionnaire was developed as a Chinese contextualized item set rather than as a verbatim translation of a single standardized foreign-language instrument, no formal translation-back-translation procedure was used. A clean reproduction of the administered Chinese questionnaire and an English translation are provided as Supplementary materials.

Role overload was measured with six items concerning work pace, amount of work, effort required to complete tasks, time pressure, rushing, and difficulty keeping up with workload. These items capture quantitative overload rather than the broader domain of role stress. Their content overlaps established role overload formulations centered on excessive work volume and insufficient time (Bolino and Turnley, 2005) and is close to the operationalization of quantitative workload emphasizing pace and amount of work (Spector and Jex, 1998). Accordingly, all substantive interpretations in this study are restricted to the quantitative workload/time–pressure facet of role overload. Job autonomy was measured with four items addressing independence in how work is carried out, freedom in work methods, the ability to adjust working time, and freedom from excessive procedural constraints; this content is consistent with method and scheduling autonomy in work-design research (Morgeson and Humphrey, 2006).

Job self-efficacy was measured with three work-domain capability items addressing the ability to perform one’s job, professional competence in one’s work domain, and confidence in one’s work capability. Their source lineage is consistent with that of job-related self-efficacy measurement (Riggs et al., 1994). Work engagement was assessed using three contextualized items addressing energy at work, willingness to go to work, and enthusiasm for work. The items are based on the short form of the Utrecht Work Engagement Scale (UWES) (Schaufeli et al., 2006) and primarily represent vigor, enthusiasm, and dedication. Job satisfaction was assessed with six contextualized favorable work-evaluation items covering overall satisfaction, sense of accomplishment, work interest, job–ability fit, identification with the organization, and pleasure in working for the current organization. The resulting score is interpreted as a contextualized job-satisfaction composite. Item wording, scoring, source lineage, and item-level standardized loadings are reported in Supplementary Tables S1C,D.

### 3.3 Statistical analysis

Analyses were conducted in R/RStudio and included measurement model evaluation, multi-group measurement invariance testing by occupational role, descriptive statistics and correlations, total-association models, mediation analysis, first-stage moderation, joint models, and sensitivity analyses. Confirmatory factor analysis (CFA) evaluated the five-factor measurement structure using the chi-square statistic (χ^2^), χ^2^/df, Root Mean Square Error of Approximation (RMSEA), Comparative Fit Index (CFI), Tucker–Lewis Index (TLI), and Standardized Root Mean Square Residual (SRMR). Internal consistency was assessed with Cronbach’s α and composite reliability (CR), convergent validity with average variance extracted (AVE), and discriminant validity with the Fornell–Larcker criterion and heterotrait–monotrait ratio (HTMT). Analysis scripts, the variable codebook, and key outputs are provided in the reproducibility materials.

All focal variables were measured by self-report at one time point, so common-method variance was examined using complementary CFA-based sensitivity analyses (Podsakoff et al., 2003, 2012). The theoretical five-factor model was compared with a one-factor CFA, an equal-loading general-method-factor model, and an unrestricted orthogonal general-factor model. The questionnaire did not include an independent marker variable. Supplementary Tables S2A,B report model fit, the unrestricted general-factor loading pattern, substantive loadings, standardized residual variances, and improper-solution diagnostics.

Because the sample included coaches, athletes, and referees/technical-support personnel, multi-group CFA using maximum-likelihood estimation tested whether the five focal constructs had comparable measurement properties across occupational groups. Configural, metric, scalar, and strict invariance were examined sequentially, with ΔCFI, ΔRMSEA, and ΔSRMR used to judge substantive deterioration after adding equality constraints (Chen, 2007; Putnick and Bornstein, 2016). Occupational-role dummy variables were then added to the conditional-process models to test whether the focal interaction and moderated indirect association were sensitive to occupational composition. Full results appear in Supplementary Tables S7, S8.

All regression models report unstandardized coefficients (B). Because heteroskedasticity was present in the primary equations, inference used HC3 heteroskedasticity-robust standard errors (SEs) and 95% confidence intervals. Numerical *p*-values are reported to three decimals when *p* ≥ 0.001; smaller values are reported as *p* < 0.001. Primary models were adjusted for sex, with male participants as the reference category. H1a and H1b tested the total associations of role overload and job autonomy with job satisfaction. H2a and H2b used regression equations corresponding to PROCESS Model 4 to estimate indirect associations through work engagement. Let X denote role overload (RO) or job autonomy (JA), M denote work engagement (WE), Y denote job satisfaction (JS), and C denote the sex covariate:


$$M_{i}=a_{0}+a_{1}X_{i}+\gamma^{T}C_{i}+\varepsilon M_{i}$$ (1)

$$Y_{i}=b_{0}+c^{\prime }X_{i}+b_{1}M_{i}+\delta^{T}C_{i}+\varepsilon Y_{i}$$ (2)

$$\mathrm{IE}=a_{1}b_{1}$$ (3)

Here, a₁ represents the association between X and M, b₁ the association between M and Y conditional on X, c′ the direct association between X and Y, and a₁b₁ the indirect association through work engagement. Role overload and job autonomy were also entered simultaneously in a joint mediation model to evaluate their independent pathways. A supplementary sensitivity analysis additionally adjusted for the archived six-level anonymous category code; because its semantic labels are unavailable, the code is not interpreted substantively.

H3 and H4 were estimated using regression equations corresponding to PROCESS Model 7 (Hayes, 2017). Role overload, job autonomy, and job self-efficacy were mean-centered before interaction terms were created, and job self-efficacy was specified as the first-stage moderator. Let W denote job self-efficacy (JSE), and subscript c denote mean-centered variables:


$$M_{i}=a_{0}+a_{1}Xc_{i}+a_{2}Wc_{i}+a_{3}(Xc_{i}\times Wc_{i})+\gamma^{T}C_{i}+\varepsilon M_{i}$$ (4)

$$\mathrm{IE}(w)=(a₁+a_{3}w)b₁;\text{IndexMM}=a_{3}b_{1}$$ (5)

IE(w) is the conditional indirect association at a specified level w of job self-efficacy, and IndexMM is the index of moderated mediation. Conditional indirect associations were evaluated at the sample mean and at ±1 SD of job self-efficacy. Indirect associations and indices of moderated mediation were estimated using 10,000 non-parametric bootstrap resamples with a fixed random seed of 20,260,912.

To evaluate whether the role-overload and job-autonomy pathways remained independent within one statistical framework, a joint moderation model was additionally estimated:


$$\begin{array}{l} M_{i}=a_{0}+a_{1}\mathrm{RO}C_{i}+a_{2}\mathrm{JA}c_{i}+a_{3}\mathrm{JSE}c_{i}\\ +a_{4}(\mathrm{RO}c_{i}\times \mathrm{JSE}c_{i})+a_{5}(\mathrm{JA}c_{i}\times \mathrm{JSE}c_{i})\\ +\gamma^{T}C_{i}+\varepsilon M_{i} \end{array}$$ (6)

$$Y_{i}=b_{0}+c_{1}^{\prime }\mathrm{RO}c_{i}+c_{2}^{\prime }\mathrm{JA}c_{i}+b_{1}M_{i}+\delta^{T}C_{i}+\varepsilon Y_{i}$$ (7)

The mediation, moderated-mediation, and joint conditional-process models are specified in Equation 1–7.

Figure 2 uses the Johnson–Neyman procedure to identify the region in which the conditional association between role overload and work engagement becomes statistically distinguishable from zero across levels of job self-efficacy. Full equations, coefficients, and computational details are reported in Supplementary Tables S3–S5, S7–S8.

**Figure 2:**
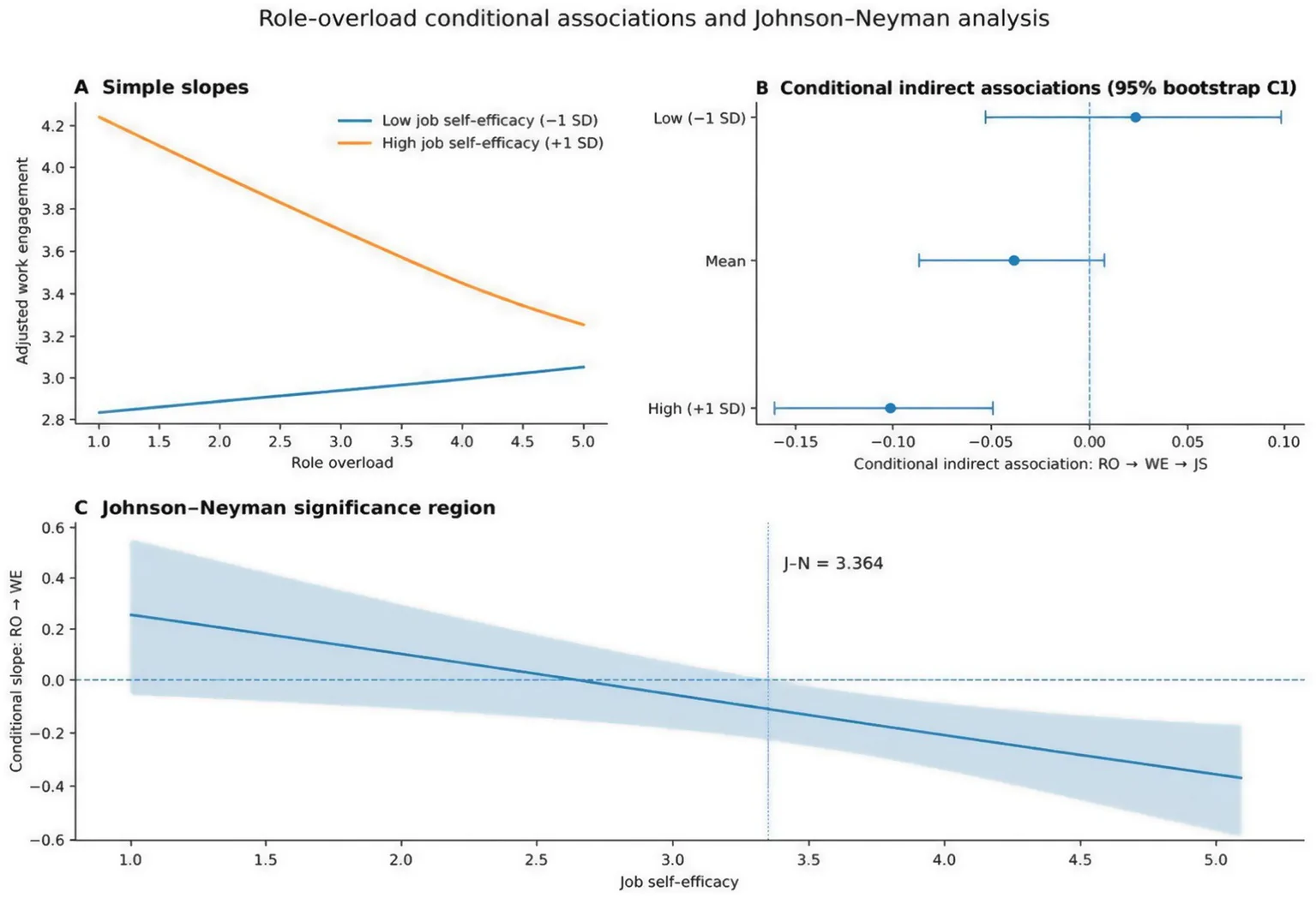
Conditional associations in the role-overload pathway and Johnson–Neyman analysis. Panel **(A)** shows sex-adjusted simple slopes from the primary Model 7 at low (−1 SD) and high (+1 SD) job self-efficacy. Panel **(B)** shows the conditional RO → WE → JS indirect associations and 95% bootstrap CIs at low, mean, and high job self-efficacy. Panel **(C)** shows the continuous conditional RO → WE slope and its 95% CI; the Johnson–Neyman threshold is 3.364.

Model diagnostics included variance inflation factors (VIFs), Cook’s distance, externally studentized residuals, and tests of heteroskedasticity. In the joint moderation model, the maximum VIF was 1.233, the maximum Cook’s distance was 0.035, and the largest absolute externally studentized residual was 2.725; no single influential observation dominated the findings. The Breusch–Pagan test indicated heteroskedasticity (*p* < 0.001), supporting the use of HC3 robust standard errors for primary inference.

## 4 Results

### 4.1 Measurement model, reliability, validity, and common-method sensitivity

The five-factor measurement model showed good fit, χ^2^ = 382.939, df = 199, χ^2^/df = 1.924, RMSEA = 0.051, CFI = 0.952, TLI = 0.944, and SRMR = 0.058 (Table 2). Item-level standardized loadings ranged from 0.700 to 0.872 and are reported individually in Supplementary Table S1D. Across the five constructs, Cronbach’s α ranged from 0.795 to 0.892, CR from 0.803 to 0.893, and AVE from 0.556 to 0.589. For each construct, the square root of AVE exceeded its absolute correlations with the other constructs, and HTMT values ranged from 0.232 to 0.659. Together, these indices supported the internal consistency and empirical separation of the five focal scores.

**Table 2:** Reliability and convergent validity of the measurement model.

Construct	Items	α	Std. loading	CR	AVE	√AVE
Role overload (RO)	6	0.880	0.700–0.872	0.882	0.556	0.745
Job autonomy (JA)	4	0.850	0.725–0.812	0.851	0.589	0.767
Job self-efficacy (JSE)	3	0.795	0.724–0.816	0.803	0.576	0.759
Work engagement (WE)	3	0.807	0.758–0.772	0.808	0.584	0.764
Job satisfaction (JS)	6	0.892	0.713–0.837	0.893	0.583	0.763

α = Cronbach’s α; CR = composite reliability; AVE = average variance extracted. The Std. loading column shows construct-level ranges for compactness; the 22 item-level standardized loadings are reported in Supplementary Table S1D.

The one-factor CFA fit substantially worse than the five-factor model (χ^2^/df = 9.737, RMSEA = 0.156, CFI = 0.525, TLI = 0.475, SRMR = 0.140). The equal-loading general-method-factor model produced essentially no improvement. The unrestricted orthogonal general-factor sensitivity model improved fit substantially (χ^2^ = 215.352, df = 177, RMSEA = 0.025, CFI = 0.990, TLI = 0.987, SRMR = 0.029). Its standardized general-factor loadings were negative for all six role-overload items (−0.261 to −0.458) and positive for all 16 job-autonomy, job-self-efficacy, work-engagement, and job-satisfaction items (0.362 to 0.622), indicating a strong evaluative-polarity pattern. No Heywood-type improper estimate was evident: standardized residual variances were all positive (0.095–0.515), and no absolute standardized loading exceeded 1. The unrestricted model therefore indicates a substantial general evaluative component but does not permit a unique attribution to method variance. Full loadings and diagnostics are reported in Supplementary Table S2B.

Multi-group CFA further evaluated measurement comparability across occupational roles. The three-group configural model showed acceptable fit (χ^2^ = 835.968, df = 597, CFI = 0.938, RMSEA = 0.034, SRMR = 0.073). Relative to the configural model, constraining factor loadings to equality yielded ΔCFI = −0.001 and ΔRMSEA = −0.001; imposing equal intercepts produced a further ΔCFI = −0.002 and ΔRMSEA = −0.001. The strict model likewise showed no substantive deterioration, and χ^2^ difference tests were non-significant at the metric, scalar, and strict stages. These results supported metric, scalar, and strict measurement invariance across occupational roles and indicated adequate measurement comparability for the pooled analyses.

### 4.2 Descriptive statistics and associations among variables

The descriptive pattern was consistent with the dual-path expectations (Table 3). Role overload correlated negatively with work engagement (*r* = −0.188) and job satisfaction (r = −0.329). Job autonomy correlated positively with work engagement (r = 0.225) and job satisfaction (r = 0.293). Job self-efficacy correlated positively with work engagement (r = 0.446) and job satisfaction (r = 0.550), and the correlation between work engagement and job satisfaction was 0.486. The largest absolute correlation among the five constructs was 0.550, indicating meaningful relationships without empirical redundancy.

**Table 3:** Descriptive statistics, correlations, and discriminant validity (*N* = 358).

Variable	M	SD	1	2	3	4	5
1. Role overload	2.696	0.899	0.745				
2. Job autonomy	3.309	0.972	−0.267**	0.767			
3. Job self-efficacy	3.266	0.982	−0.242**	0.286**	0.759		
4. Work engagement	3.413	1.033	−0.188**	0.225**	0.446**	0.764	
5. Job satisfaction	3.264	0.968	−0.329**	0.293**	0.550**	0.486**	0.763

Diagonal values are √AVE; the lower triangle contains Pearson correlations. **p < 0.01, two-tailed.

### 4.3 Main associations and indirect associations through work engagement

After adjustment for sex (Table 4), role overload was negatively associated with job satisfaction (B = −0.355, HC3 SE = 0.051, 95% CI [−0.454, −0.256], *p* < 0.001), whereas job autonomy was positively associated with job satisfaction (B = 0.293, HC3 SE = 0.048, 95% CI [0.198, 0.388], *p* < 0.001). When role overload and job autonomy were entered simultaneously in the total-association model, both retained independent associations: B = −0.291 (*p* < 0.001) for role overload and B = 0.221 (*p* < 0.001) for job autonomy. H1a and H1b were supported.

**Table 4:** Primary regression and first-stage moderation results.

Test	Path	B	HC3 SE	*p*	95% CI
H1a total association	RO → JS	−0.355	0.051	< 0.001	[−0.454, −0.256]
H1b total association	JA → JS	0.293	0.048	< 0.001	[0.198, 0.388]
H2a a path	RO → WE	−0.216	0.058	< 0.001	[−0.330, −0.102]
H2a b path	WE → JS (RO model)	0.415	0.043	< 0.001	[0.330, 0.500]
H2b a path	JA → WE	0.238	0.055	< 0.001	[0.131, 0.346]
H2b b path	WE → JS (JA model)	0.418	0.043	< 0.001	[0.333, 0.502]
H3a moderation	RO × JSE → WE	−0.152	0.057	0.008	[−0.265, −0.040]
H3b moderation	JA × JSE → WE	0.106	0.057	0.063	[−0.006, 0.218]

B = unstandardized coefficient; HC3 SE = heteroskedasticity-robust standard errors. All primary regressions adjust for sex (male reference). Interaction terms were constructed from mean-centered variables.

Work engagement showed stable indirect associations in both pathways. In the role-overload model, role overload was negatively associated with work engagement (B = −0.216, *p* < 0.001), and work engagement was positively associated with job satisfaction (B = 0.415, *p* < 0.001); the bootstrap indirect effect was −0.090, 95% CI [−0.141, −0.043]. In the job-autonomy model, job autonomy was positively associated with work engagement (B = 0.238, *p* < 0.001), and work engagement was positively associated with job satisfaction (B = 0.418, *p* < 0.001); the indirect effect was 0.100, 95% CI [0.055, 0.148]. H2a and H2b were supported.

When role overload and job autonomy were entered simultaneously in a joint mediation model, both indirect associations remained. The independent association of role overload with work engagement was B = −0.158 (*p* = 0.012), the independent association of job autonomy with work engagement was B = 0.199 (*p* < 0.001), and the association of work engagement with job satisfaction was B = 0.391 (*p* < 0.001). The corresponding unique indirect effects were −0.062, 95% CI [−0.114, −0.014], for role overload and 0.078, 95% CI [0.033, 0.128], for job autonomy.

### 4.4 Conditional role of job self-efficacy

The role-overload pathway showed a consistent conditional pattern (Table 5). The role overload × job self-efficacy interaction was negative (B = −0.152, HC3 SE = 0.057, 95% CI [−0.265, −0.040], *p* = 0.008). The conditional indirect effect was 0.023, 95% CI [−0.053, 0.097], at low job self-efficacy (−1 SD), −0.039, 95% CI [−0.087, 0.006], at the mean, and −0.101, 95% CI [−0.161, −0.051], at high job self-efficacy (+1 SD). The index of moderated mediation was −0.063, 95% CI [−0.111, −0.016], supporting H3a and H4a. Johnson–Neyman analysis indicated that the adjusted association between role overload and work engagement was significantly negative when job self-efficacy was above 3.36. The corresponding simple slopes, conditional indirect associations, and Johnson–Neyman region are shown in Figure 2.

**Table 5:** Conditional indirect associations and indices of moderated mediation.

Path	JSE level/Index	Effect	Boot SE	95% Boot CI
RO → WE → JS	Low JSE (−1 SD)	0.023	0.038	[−0.053, 0.097]
RO → WE → JS	Mean JSE	−0.039	0.023	[−0.087, 0.006]
RO → WE → JS	High JSE (+1 SD)	−0.101	0.028	[−0.161, −0.051]
RO pathway	Index of moderated mediation	−0.063	0.024	[−0.111, −0.016]
JA → WE → JS	Low JSE (−1 SD)	0.003	0.037	[−0.071, 0.075]
JA → WE → JS	Mean JSE	0.047	0.023	[0.002, 0.093]
JA → WE → JS	High JSE (+1 SD)	0.091	0.029	[0.036, 0.152]
JA pathway	Index of moderated mediation	0.044	0.024	[−0.001, 0.093]
Joint RO pathway	Index of moderated mediation	−0.049	0.024	[−0.096, −0.003]
Joint JA pathway	Index of moderated mediation	0.029	0.023	[−0.016, 0.075]

Bootstrap = 10,000 resamples with random seed 20,260,912. Job self-efficacy levels are the sample mean and mean ±1 SD. Primary models adjust for sex (male reference).

The job-autonomy pathway showed a different pattern. Positive associations of job autonomy with work engagement and job satisfaction remained stable, whereas the job autonomy × job self-efficacy interaction was not statistically supported (B = 0.106, HC3 SE = 0.057, 95% CI [−0.006, 0.218], *p* = 0.063). The corresponding index of moderated mediation was 0.044, 95% CI [−0.001, 0.093]. H3b and H4b were therefore not supported.

The joint moderation model reinforced this asymmetry. The role overload × job self-efficacy interaction remained negative (B = −0.124, HC3 SE = 0.060, 95% CI [−0.242, −0.007], *p* = 0.038), with a corresponding index of moderated mediation of −0.049, 95% CI [−0.096, −0.003]. The job autonomy × job self-efficacy interaction was B = 0.073 (*p* = 0.228), and its index of moderated mediation was 0.029, 95% CI [−0.016, 0.075]. Results were materially unchanged in the supplementary model that adjusted for the archived anonymous six-level category code.

Occupational-role sensitivity analyses yielded the same pattern of inference. With coach as the reference group and dummy variables for athlete and referee/technical-support personnel added to the Model 7 analysis, RO × JSE remained negative and significant (B = −0.151, HC3 SE = 0.058, 95% CI [−0.264, −0.037], *p* = 0.009), and the corresponding index of moderated mediation was −0.062, 95% bootstrap CI [−0.110, −0.015]. In the role-adjusted joint model, RO × JSE remained significant (B = −0.123, *p* = 0.042), with a joint RO index of moderated mediation of −0.048, 95% CI [−0.095, −0.002]. JA × JSE and its moderated-mediation indices remained unsupported. The focal role-overload conditional pattern was robust to adjustment for occupational-role composition.

## 5 Discussion

The central empirical result was an asymmetric conditional pattern. Role overload was negatively associated with job satisfaction, job autonomy was positively associated with job satisfaction, and work engagement formed an indirect association in both pathways. Higher job self-efficacy was associated with a stronger negative role overload–work engagement association, whereas the positive job-autonomy pathway was expressed primarily as a stable independent association. This asymmetry is consistent with a context-dependent interpretation of personal-resource functioning and directs attention to the work conditions under which capability beliefs may show different associations with engagement.

### 5.1 Sport work as a two-dimensional structure of demand intensity and action control

The joint models showed that role overload and job autonomy retained independent associations in opposite directions after adjustment for one another. This pattern supports distinguishing two non-interchangeable work-design dimensions in sport settings: demand intensity and action control. Role overload captures perceived quantitative workload intensity and time pressure, including difficulty keeping up with current workload; job autonomy captures discretion over work processes, methods, and scheduling. High demands do not necessarily imply low autonomy, and low demands do not imply high autonomy. Treating sport work as a single continuum of high versus low stress would therefore obscure a functionally important distinction.

Work engagement was statistically associated with both demand intensity and action control in the expected directions. Role overload was associated with lower job satisfaction through lower work engagement, whereas job autonomy was associated with higher job satisfaction through higher work engagement, and both unique indirect associations remained in the joint model. This pattern is broadly consistent with longitudinal JD–R research and meta-analytic evidence on work engagement (Lesener et al., 2019; Mazzetti et al., 2023). In the present cross-sectional study, these indirect associations represent statistical pathways rather than evidence of temporal mediation.

### 5.2 Job self-efficacy and the contextual boundary of personal resource translation

The role-overload pathway produced the most distinctive result. In the primary Model 7, the RO × JSE interaction was significantly negative, and the bootstrap interval for the index of moderated mediation excluded zero. The interaction retained the same direction and inferential classification in the joint model, and Johnson–Neyman analysis showed that the negative role overload–work engagement association became statistically significant when job self-efficacy was above 3.36. The direction of the interaction differed from the common buffering hypothesis for self-efficacy: role overload was more clearly associated with lower work engagement at higher levels of job self-efficacy.

Ability-control mismatch offers one possible interpretation of the role-overload interaction. Job self-efficacy reflects confidence in one’s ability to perform and persist, whereas the present role-overload indicator captures workload and time–pressure demands. Strong capability beliefs may be less readily translated into favorable work experience when high quantitative demands cannot be altered through individual action. Conservation of Resources Theory provides a conceptual basis for this possibility because resource investment is expected to be more sustainable when it protects resources or generates gains (Halbesleben et al., 2014; Hobfoll et al., 2018). Future studies can test this explanation by directly measuring task controllability, effort investment, recovery, staffing, and capacity to alter workload.

The findings also caution against assuming that job self-efficacy uniformly buffers job demands. Xanthopoulou et al. (2007) similarly found that personal resources did not consistently weaken adverse demand associations, and Jex and Bliese (1999) and Schaubroeck et al. (2001) demonstrated context dependence in relations between efficacy beliefs and work stress. The present pattern is therefore consistent with the proposition that associations involving personal resources may vary across work structures. Direct tests of that proposition require measures of controllability, resource investment, recovery, and temporal change.

### 5.3 Job autonomy and the conditional role of job self-efficacy

The job-autonomy pathway showed a different statistical pattern. Job autonomy provides measured latitude in method choice, task scheduling, and professional judgment, and its positive associations with work engagement and job satisfaction remained stable across models. The JSE × JA interaction was not statistically supported, whereas the positive associations of job autonomy and job self-efficacy with work engagement remained. Future research can examine whether functional overlap in perceived control helps explain this pattern.

The autonomy results therefore did not provide evidence that the association of job autonomy with work engagement varied across levels of job self-efficacy. Job autonomy and job self-efficacy each showed positive associations with work engagement, but their interaction was not robustly supported. This contrast with the role-overload pathway helps define a more specific question for future work: when does a personal resource add independently to a job resource, and when does it alter the association between work structure and engagement?

### 5.4 Theoretical contributions

The study contributes to JD–R research by documenting an asymmetric conditional association across two contrasting work characteristics. Existing research has established associations between personal resources and work engagement but has paid less attention to whether such associations vary across specific demand and control conditions. The present findings provide a basis for more direct tests of context-dependent personal-resource functioning.

The framework also separates three functions that are often discussed together: capability belief, action control, and quantitative demand intensity. Job self-efficacy captures confidence in work capability; job autonomy captures discretion over how work is performed; and the role-overload measure captures workload and time–pressure intensity. Their distinct associations indicate that workload, autonomy, and efficacy should not be treated as interchangeable indicators of general positive or negative work conditions.

The asymmetry itself provides the most specific theoretical proposition. The role-overload pathway showed a negative conditional association, whereas the job-autonomy pathway showed stable independent associations without a robust incremental interaction. Future research can test whether the association of personal resources with engagement depends on task controllability and on whether additional effort produces recoverable gains, using direct measures of controllability, objective workload, effort investment, recovery, and effort-outcome correspondence.

### 5.5 Practical implications

At the practical level, quantitative workload and time pressure emerge as work characteristics that merit systematic monitoring. Higher role-overload scores were associated with lower work engagement and job satisfaction and with a stronger negative role–overload–work engagement association at higher job self-efficacy. In sport organizations, workload review can therefore focus on task volume, deadlines, unplanned task demands, staffing, and the match between responsibilities and available resources.

Job autonomy represents a separate work-design target. Its stable positive associations with work engagement and job satisfaction suggest value in examining whether work methods, scheduling, and on-site professional judgment are appropriately aligned with role responsibilities. Any redesign should be evaluated prospectively so that changes in work structure can be distinguished from pre-existing differences among personnel.

The job self-efficacy findings suggest that personal-resource development should be considered alongside work-design conditions. The occupational relevance of capability beliefs may depend on whether employees have sufficient latitude and resources to act on those beliefs. Future intervention studies can test whether addressing individual capability together with structural work conditions yields greater improvements than personal-resource development alone.

### 5.6 Limitations and future research

Several limitations define the scope of inference. The cross-sectional design precludes temporal and causal interpretation of the indirect associations. The focal framework was developed *post hoc* following preliminary empirical inspection, which limits confirmatory inference and increases the importance of independent replication. Preregistered longitudinal or experimental designs are needed to test the conditional pattern prospectively.

All focal variables were self-reported. The five-factor model fit substantially better than the one-factor model, but the unrestricted general-factor sensitivity model also improved fit substantially. Its general-factor loadings followed the evaluative polarity of the items, and no Heywood-type improper estimate was evident; nevertheless, the factor cannot be identified as pure method variance. Residual common-method variance and general evaluative covariance may therefore have influenced association magnitudes. Future studies should combine self-report measures with supervisor ratings, objective workload, recorded working hours, scheduling data, or other sources.

The sample was recruited non-probabilistically, and a conventional response rate could not be calculated because the denominator of questionnaires distributed across multiple offline channels was not retained. Adult status was verified from identity documents before participation, but age was not recorded as a numeric analytic variable; education, employment status, and occupational tenure were also not collected. Primary sport and broad region were available only as aggregate paper tallies without row-level linkage to outcomes, so participant-level demographic reporting is limited to sex and occupational role. Multi-group CFA supported measurement invariance across coaches, athletes, and referees/technical-support personnel, and role-adjusted sensitivity analyses did not materially alter the focal RO × JSE pattern. Measurement limitations also remain: the role-overload indicator is restricted to quantitative workload/time pressure; job self-efficacy and work engagement use narrow three-item contextualized forms; and the job-satisfaction composite includes job-ability fit and organizational-identification content in addition to direct satisfaction items. Replication with independently validated full-length or established short-form measures would strengthen construct coverage. Task controllability, effort investment, resource recovery, staffing, and the capacity to alter workload were not measured directly; their roles therefore remain hypotheses for prospective testing.

## 6 Conclusion

This study identified distinct associations of quantitative role overload and job autonomy with work engagement and job satisfaction among sports practitioners. Work engagement formed an indirect association in both pathways, and higher job self-efficacy was associated with a more negative role overload–work engagement association, whereas the job-autonomy interaction with job self-efficacy was not robustly supported. This asymmetry is consistent with a context-sensitive account of personal-resource functioning and provides a focused hypothesis for longitudinal and experimental tests of whether associations involving capability beliefs differ across work structures.

## Data Availability

The original contributions presented in the study are included in the article/Supplementary material, further inquiries can be directed to the corresponding author(s).

## References

[ref1] Arnold R. Collington S. Manley H. Rees S. Soanes J. Williams M. (2019). “The team behind the team”: exploring the organizational stressor experiences of sport science and management staff in elite sport. J. Appl. Sport Psychol. 31, 7–26. doi: 10.1080/10413200.2017.1407836

[ref2] Arnold R. Fletcher D. (2012). A research synthesis and taxonomic classification of the organizational stressors encountered by sport performers. J. Sport Exerc. Psychol. 34, 397–429. doi: 10.1123/jsep.34.3.397 22691400

[ref3] Bakker A. B. Demerouti E. (2007). The job demands-resources model: state of the art. J. Manager. Psychol. 22, 309–328. doi: 10.1108/02683940710733115

[ref4] Bakker A. B. Demerouti E. (2017). Job demands-resources theory: taking stock and looking forward. J. Occup. Health Psychol. 22, 273–285. doi: 10.1037/ocp0000056, 27732008

[ref5] Bakker A. B. Demerouti E. Sanz-Vergel A. I. (2014). Burnout and work engagement: the JD-R approach. Annu. Rev. Organ. Psychol. Organ. Behav. 1, 389–411. doi: 10.1146/annurev-orgpsych-031413-091235

[ref6] Bandura A. (1997). Self-Efficacy: The Exercise of Control. New York: W. H. Freeman.

[ref7] Bolino M. C. Turnley W. H. (2005). The personal costs of citizenship behavior: the relationship between individual initiative and role overload, job stress, and work-family conflict. J. Appl. Psychol. 90, 740–748. doi: 10.1037/0021-9010.90.4.740, 16060790

[ref8] Chen F. F. (2007). Sensitivity of goodness of fit indexes to lack of measurement invariance. Struct. Equ. Model. 14, 464–504. doi: 10.1080/10705510701301834

[ref9] Christian M. S. Garza A. S. Slaughter J. E. (2011). Work engagement: a quantitative review and test of its relations with task and contextual performance. Pers. Psychol. 64, 89–136. doi: 10.1111/j.1744-6570.2010.01203.x

[ref10] Clausen T. Pedersen L. R. M. Andersen M. F. Theorell T. Madsen I. E. H. (2022). Job autonomy and psychological well-being: a linear or a non-linear association? Eur. J. Work Organ. Psychol. 31, 395–405. doi: 10.1080/1359432X.2021.1972973

[ref11] Crawford E. R. LePine J. A. Rich B. L. (2010). Linking job demands and resources to employee engagement and burnout: a theoretical extension and meta-analytic test. J. Appl. Psychol. 95, 834–848. doi: 10.1037/a0019364, 20836586

[ref12] de Jonge J. Dormann C. (2006). Stressors, resources, and strain at work: a longitudinal test of the triple-match principle. J. Appl. Psychol. 91, 1359–1374. doi: 10.1037/0021-9010.91.5.1359, 17100490

[ref13] Demerouti E. Bakker A. B. Nachreiner F. Schaufeli W. B. (2001). The job demands-resources model of burnout. J. Appl. Psychol. 86, 499–512. doi: 10.1037/0021-9010.86.3.499, 11419809

[ref14] Guo Y. Chang P.-C. Liu S. Cai Q. (2025). How role overload impacts nurses’ work engagement in healthcare emergencies: a moderated mediation model. Int. Nurs. Rev. 72:e70125. doi: 10.1111/inr.70125, 41165065

[ref15] Hagger M. S. McAnally Star K. (2026). Self-determination theory and workplace outcomes: a meta-analysis. Stress. Health 42:e70151. doi: 10.1002/smi.70151, 41664590 PMC12887546

[ref16] Hakanen J. J. Schaufeli W. B. Ahola K. (2008). The job demands-resources model: a three-year cross-lagged study of burnout, depression, commitment, and work engagement. Work Stress 22, 224–241. doi: 10.1080/02678370802379432

[ref17] Halbesleben J. R. B. Neveu J.-P. Paustian-Underdahl S. C. Westman M. (2014). Getting to the “COR”: understanding the role of resources in conservation of resources theory. J. Manag. 40, 1334–1364. doi: 10.1177/0149206314527130

[ref18] Hayes A. F. (2017). Introduction to Mediation, Moderation, and Conditional process Analysis: A Regression-based Approach. New York: Guilford Press.

[ref19] Hobfoll S. E. (1989). Conservation of resources: a new attempt at conceptualizing stress. Am. Psychol. 44, 513–524. doi: 10.1037/0003-066X.44.3.513, 2648906

[ref20] Hobfoll S. E. (2002). Social and psychological resources and adaptation. Rev. Gen. Psychol. 6, 307–324. doi: 10.1037/1089-2680.6.4.307

[ref21] Hobfoll S. E. Halbesleben J. Neveu J.-P. Westman M. (2018). Conservation of resources in the organizational context: the reality of resources and their consequences. Annu. Rev. Organ. Psychol. Organ. Behav. 5, 103–128. doi: 10.1146/annurev-orgpsych-032117-104640

[ref22] Jex S. M. Bliese P. D. (1999). Efficacy beliefs as a moderator of the impact of work-related stressors: a multilevel study. J. Appl. Psychol. 84, 349–361. doi: 10.1037/0021-9010.84.3.349, 10380416

[ref23] Kahn W. A. (1990). Psychological conditions of personal engagement and disengagement at work. Acad. Manag. J. 33, 692–724. doi: 10.2307/256287

[ref24] Kahn R. L. Wolfe D. M. Quinn R. P. Snoek J. D. Rosenthal R. A. (1964). Organizational Stress: Studies in Role Conflict and Ambiguity. New York: Wiley.

[ref25] Karasek R. A. (1979). Job demands, job decision latitude, and mental strain: implications for job redesign. Adm. Sci. Q. 24, 285–308. doi: 10.2307/2392498

[ref26] Kaski S. Kinnunen U. (2021). Work-related ill- and well-being among Finnish sport coaches: exploring the relationships between job demands, job resources, burnout and work engagement. Int. J. Sports Sci. Coach. 16, 262–271. doi: 10.1177/1747954120967794

[ref27] Lesener T. Gusy B. Wolter C. (2019). The job demands-resources model: a meta-analytic review of longitudinal studies. Work Stress. 33, 76–103. doi: 10.1080/02678373.2018.1529065

[ref28] Llorens S. Schaufeli W. Bakker A. Salanova M. (2007). Does a positive gain spiral of resources, efficacy beliefs and engagement exist? Comput. Hum. Behav. 23, 825–841. doi: 10.1016/j.chb.2004.11.012

[ref29] Mazzetti G. Robledo E. Vignoli M. Topa G. Guglielmi D. Schaufeli W. B. (2023). Work engagement: a meta-analysis using the job demands-resources model. Psychol. Rep. 126, 1069–1107. doi: 10.1177/00332941211051988, 34886729

[ref30] Mercer R. A. Russell J. L. Strack D. S. Coutts A. J. McLean B. D. (2025). Work demands, responses, and coping strategies for staff in high-performance sport: a scoping review. Sports Med. 55, 3001–3058. doi: 10.1007/s40279-025-02324-0, 41028555

[ref31] Morgeson F. P. Humphrey S. E. (2006). The work design questionnaire (WDQ): developing and validating a comprehensive measure for assessing job design and the nature of work. J. Appl. Psychol. 91, 1321–1339. doi: 10.1037/0021-9010.91.6.1321, 17100487

[ref32] Orgambídez A. Extremera N. (2020). Understanding the link between work engagement and job satisfaction: do role stressors underlie this relationship? Scand. J. Psychol. 61, 443–449. doi: 10.1111/sjop.12613, 31968141

[ref33] Podsakoff P. M. MacKenzie S. B. Lee J.-Y. Podsakoff N. P. (2003). Common method biases in behavioral research: a critical review of the literature and recommended remedies. J. Appl. Psychol. 88, 879–903. doi: 10.1037/0021-9010.88.5.879, 14516251

[ref34] Podsakoff P. M. MacKenzie S. B. Podsakoff N. P. (2012). Sources of method bias in social science research and recommendations on how to control it. Annu. Rev. Psychol. 63, 539–569. doi: 10.1146/annurev-psych-120710-100452, 21838546

[ref35] Potts A. J. Didymus F. F. Kaiseler M. (2023). Psychological stress and psychological well-being among sports coaches: a meta-synthesis of the qualitative research evidence. Int. Rev. Sport Exerc. Psychol. 16, 554–583. doi: 10.1080/1750984X.2021.1907853

[ref36] Putnick D. L. Bornstein M. H. (2016). Measurement invariance conventions and reporting: the state of the art and future directions for psychological research. Dev. Rev. 41, 71–90. doi: 10.1016/j.dr.2016.06.004, 27942093 PMC5145197

[ref37] Rich B. L. LePine J. A. Crawford E. R. (2010). Job engagement: antecedents and effects on job performance. Acad. Manag. J. 53, 617–635. doi: 10.5465/amj.2010.51468988

[ref38] Riggs M. L. Warka J. Babasa B. Betancourt R. Hooker S. (1994). Development and validation of self-efficacy and outcome expectancy scales for job-related applications. Educ. Psychol. Meas. 54, 793–802. doi: 10.1177/0013164494054003026

[ref39] Rizzo J. R. House R. J. Lirtzman S. I. (1970). Role conflict and ambiguity in complex organizations. Adm. Sci. Q. 15, 150–163. doi: 10.2307/2391486

[ref40] Salanova M. Llorens S. Schaufeli W. B. (2011). “Yes, I can, I feel good, and I just do it!” on gain cycles and spirals of efficacy beliefs, affect, and engagement. Appl. Psychol. 60, 255–285. doi: 10.1111/j.1464-0597.2010.00435.x

[ref41] Schaubroeck J. Jones J. R. Xie J. L. (2001). Individual differences in utilizing control to cope with job demands: effects on susceptibility to infectious disease. J. Appl. Psychol. 86, 265–278. doi: 10.1037/0021-9010.86.2.265, 11393439

[ref42] Schaufeli W. B. Bakker A. B. Salanova M. (2006). The measurement of work engagement with a short questionnaire: a cross-national study. Educ. Psychol. Meas. 66, 701–716. doi: 10.1177/0013164405282471

[ref43] Schaufeli W. B. Salanova M. González-Romá V. Bakker A. B. (2002). The measurement of engagement and burnout: a two-sample confirmatory factor analytic approach. J. Happiness Stud. 3, 71–92. doi: 10.1023/A:1015630930326

[ref44] Simpson R. A. C. Didymus F. F. Williams T. L. (2024). Organizational stress and well-being in competitive sport: a systematic review. Int. Rev. Sport Exerc. Psychol. 17, 116–144. doi: 10.1080/1750984X.2021.1975305

[ref45] Spector P. E. (1986). Perceived control by employees: a meta-analysis of studies concerning autonomy and participation at work. Hum. Relat. 39, 1005–1016. doi: 10.1177/001872678603901104

[ref46] Spector P. E. Jex S. M. (1998). Development of four self-report measures of job stressors and strain: interpersonal conflict at work scale, organizational constraints scale, quantitative workload inventory, and physical symptoms inventory. J. Occup. Health Psychol. 3, 356–367. doi: 10.1037/1076-8998.3.4.356, 9805281

[ref47] Svensson P. G. Jeong S. Shuck B. Otto M. G. (2021). Antecedents and outcomes of employee engagement in sport for development. Sport Manag. Rev. 24, 673–696. doi: 10.1080/14413523.2021.1880758

[ref48] Xanthopoulou D. Bakker A. B. Demerouti E. Schaufeli W. B. (2007). The role of personal resources in the job demands-resources model. Int. J. Stress. Manag. 14, 121–141. doi: 10.1037/1072-5245.14.2.121

[ref49] Xanthopoulou D. Bakker A. B. Demerouti E. Schaufeli W. B. (2009). Reciprocal relationships between job resources, personal resources, and work engagement. J. Vocat. Behav. 74, 235–244. doi: 10.1016/j.jvb.2008.11.003

[ref50] Yildiz B. Yildiz T. (2022). A systematic review and meta-analytical synthesis of the relationship between work engagement and job satisfaction in nurses. Perspect. Psychiatr. Care 58, 3062–3078. doi: 10.1111/ppc.13068, 35383388

[ref51] Zhang N. Xu D. Li J. Xu Z. (2022). Effects of role overload, work engagement and perceived organisational support on nurses’ job performance during the COVID-19 pandemic. J. Nurs. Manag. 30, 901–912. doi: 10.1111/jonm.13598, 35293044 PMC9115180

