# Supplementary material for: Contextual boundaries of personal resource translation: dual-path associations of role overload and job autonomy with job satisfaction among sports practitioners
Source: Front Psychol. 2026 Oct 7;17:1844634. doi: 10.3389/fpsyg.2026.1844634 (PMC13682992; doi:10.3389/fpsyg.2026.1844634)
Supplement: Supplementary file 2 [file Data_Sheet_2.ZIP › Supplementary Materials/Ethics_Approval_YIT-TY-IRB-2025-07-007.pdf]

人体研究伦理审查申请表

Human Research Ethics Review Application Form

**项目名称**<br>**Project Title**	体育设施指导员工作要求、工作资源与职业心理状态的线性与非线性关联<br>Linear and nonlinear associations of job demands and resources with work-related psychological outcomes among sports facility instructors		
**项目负责人**<br>**Principal Investigator**	罗伟<br>Wei Luo	**所在单位**<br>**Institution**	盐城工学院<br>Yancheng Institute of Technology
**数据采集时间**<br>**Data Collection Period**	2025 年 8 月<br>August 2025	**申请用途**<br>**Purpose of Application**	[ ] 项目申请 [√] 发表论文 [ ] 其他：_________<br>[ ] Project application [√] Journal publication [ ] Other: _________
**研究对象**<br>**Study Participants**	[√] 人体相关研究 [ ] 动物实验<br>[√] Human-participant research [ ] Animal research		
**研究方式**<br>**Study Design**	[√] 非干预性问卷调查 [ ] 体内实验 [ ] 其他：_________<br>[√] Non-interventional questionnaire survey [ ] In vivo experiment [ ] Other: _________		
**申请简述**<br>**Study Summary**	1 研究目的：考察体育从业者的工作要求、工作资源与自我效能、工作投入和工作满意度之间的关联，并探索部分关联的函数形式。<br>1. Objective: To examine associations of job demands and job resources with self-efficacy, work engagement, and job satisfaction among sports practitioners, and to explore the functional form of selected associations.<br>2 研究设计：横断面、非干预性问卷研究。<br>2. Design: A cross-sectional, non-interventional questionnaire study.<br>3 测量内容：工作要求包括角色过载、角色模糊和角色冲突；工作资源包括社会支持、工作自主性、自我发展机会和反馈；职业心理变量包括自我效能、工作投入和工作满意度。<br>3. Measures: Job demands included role overload, role ambiguity, and role conflict. Job resources included social support, work autonomy, self-development opportunities, and feedback. Work-related psychological variables included self-efficacy, work engagement, and job satisfaction.<br>4 风险评估：研究不涉及药物、侵入性操作或行为干预，预计风险不高于日常问卷调查可能产生的轻微心理或时间负担。<br>4. Risk assessment: The study involved no medication, invasive procedure, or behavioral intervention. Anticipated risk was no greater than the minor psychological or time burden associated with routine questionnaire participation.<br>5 知情与自愿：调查实施前向参与者说明研究目的、参与方式、自愿原则及退出权利；参与者在知情和自愿基础上参与。<br>5. Informed and voluntary participation: Before completing the questionnaire, participants were informed of the study purpose, participation procedures, voluntary nature of participation, and right to withdraw.<br>6 隐私与数据保护：研究数据以去身份化方式分析，不在论文或公开材料中披露可识别个人身份的信息；数据仅用于与本研究相关的科研用途。<br>6. Privacy and data protection: Data were analyzed in de-identified form. No personally identifiable information will be disclosed in publications or public materials, and the data will be used only for research purposes consistent with this study.<br>8. 发表计划：研究结果拟用于学术论文发表。<br>8. Publication plan: Findings are intended for academic publication.		
**申请人确认**<br>**Applicant Confirmation**	本人确认以上内容真实，并同意按伦理委员会要求补充或修订材料。<br>申请人签字：____罗伟________ 日期：__2025__年 _6 月 30 日<br>I confirm that the information above is accurate and agree to provide additional information or revisions as requested by the ethics committee.<br>Applicant signature: ______luowei______ Date: _6_ / _30_ / _2025_		
**伦理委员会审查意见**<br>**Ethics Committee Review**	[√] 批准 [ ] 修改后批准 [ ] 不批准<br>审查编号：_YIT-TY-IRB-2025-07-007_ 审查日期：2025_年 6 月 30 日<br>审查意见：___________同意_______________________________<br>伦理委员会签字/盖章：________________________________<br>[√] Approved [ ] Approved after revision [ ] Not approved<br>Review No.: _YIT-TY-IRB-2025-07-007_ Review date: _6_ / __30_ / __2025__<br>Comments: ___Approved________________________________<br>Ethics committee signature/stamp: ______________________________		
