# Supplementary material for: Contextual boundaries of personal resource translation: dual-path associations of role overload and job autonomy with job satisfaction among sports practitioners
Source: Front Psychol. 2026 Oct 7;17:1844634. doi: 10.3389/fpsyg.2026.1844634 (PMC13682992; doi:10.3389/fpsyg.2026.1844634)
Supplement: Supplementary file 2 [file Data_Sheet_2.ZIP › Supplementary Materials/Questionnaire_Chinese.pdf]

# Supplementary Questionnaire

## 体育从业者工作要求、工作资源与职业心理状态调查问卷

## 空白版

尊敬的参与者：您好！感谢您在百忙之中参与本次调查。
本问卷旨在了解体育从业者在工作要求、工作资源、工作自我效能、工作投入和工作满意度等方面的真实体验。问卷没有标准答案或对错之分，请您根据目前的实际工作感受如实作答。
本调查仅用于学术研究。您的参与完全自愿，您有权拒绝参加或在填写过程中停止作答，不会因此受到任何不利影响。问卷资料将严格保密，并以去身份化形式进行统计分析，不用于研究目的以外的其他用途。本调查不提供经济报酬。
本研究已经盐城工学院伦理委员会批准（YIT-TY-IRB-2025-07-007）。如您同意参加，请在完成书面知情同意后继续填写本问卷。感谢您的理解与支持。

**填写说明：**本问卷仅用于学术研究，请根据当前真实工作体验作答。除基本信息外，所有陈述均采用 5 点 Likert 计分：1=完全不同意，2=不同意，3=一般，4=同意，5=完全同意。每题仅选择一个答案。

### 第一部分 工作要求

| 编号 | 陈述 | 1 | 2 | 3 | 4 | 5 |
|---|---|---|---|---|---|---|
| RO1 | 工作需要快速处理。 | ☐ | ☐ | ☐ | ☐ | ☐ |
| RO2 | 我要做的工作太多。 | ☐ | ☐ | ☐ | ☐ | ☐ |
| RO3 | 为了完成工作，我必须非常努力。 | ☐ | ☐ | ☐ | ☐ | ☐ |
| RO4 | 我经常在时间压力下工作。 | ☐ | ☐ | ☐ | ☐ | ☐ |
| RO5 | 我经常需要匆忙赶工。 | ☐ | ☐ | ☐ | ☐ | ☐ |
| RO6 | 我觉得自己跟不上当前的工作量。 | ☐ | ☐ | ☐ | ☐ | ☐ |
| RA1 | 对于工作中我拥有什么权限，我并不清楚。 | ☐ | ☐ | ☐ | ☐ | ☐ |
| RA2 | 我的工作任务或目标并不明确。 | ☐ | ☐ | ☐ | ☐ | ☐ |
| RA3 | 在完成工作时，我不清楚应如何分配处理时间。 | ☐ | ☐ | ☐ | ☐ | ☐ |
| RA4 | 我不清楚自己在工作中应承担哪些责任。 | ☐ | ☐ | ☐ | ☐ | ☐ |
| RA5 | 我不清楚所在单位对我有什么期望。 | ☐ | ☐ | ☐ | ☐ | ☐ |
| RA6 | 在开展工作时，我没有得到关于工作方法的明确说明。 | ☐ | ☐ | ☐ | ☐ | ☐ |
| RC1 | 我缺少完成工作所需的信息。 | ☐ | ☐ | ☐ | ☐ | ☐ |
| RC2 | 为了完成工作，我有时不得不违反所在单位的规定或政策。 | ☐ | ☐ | ☐ | ☐ | ☐ |
| RC3 | 我有时会被不同的人要求完成性质不同的工作。 | ☐ | ☐ | ☐ | ☐ | ☐ |
| RC4 | 我有时承担与本职工作无关的任务。 | ☐ | ☐ | ☐ | ☐ | ☐ |
| RC5 | 我有时会承担得不到他人认同或支持的工作。 | ☐ | ☐ | ☐ | ☐ | ☐ |

### 第二部分 工作资源

| 编号 | 陈述 | 1 | 2 | 3 | 4 | 5 |
|---|---|---|---|---|---|---|
| SS1 | 同事会关心我的情绪和感受。 | ☐ | ☐ | ☐ | ☐ | ☐ |
| SS2 | 同事会关心我遇到的问题。 | ☐ | ☐ | ☐ | ☐ | ☐ |
| SS3 | 同事会在工作相关问题上真诚地帮助我。 | ☐ | ☐ | ☐ | ☐ | ☐ |

| 编号 | 陈述 | 1 | 2 | 3 | 4 | 5 |
|---|---|---|---|---|---|---|
| SS4 | 我在良好的工作氛围中工作。 | □ | □ | □ | □ | □ |
| JA1 | 在工作推进方式上，我具有独立性。 | □ | □ | □ | □ | □ |
| JA2 | 在工作推进方式上，我有较大的自由。 | □ | □ | □ | □ | □ |
| JA3 | 我可以与同事协商调整工作时间。 | □ | □ | □ | □ | □ |
| JA4 | 工作时，我不必过度受到繁琐程序的限制。 | □ | □ | □ | □ | □ |
| FB1 | 我会获得关于自己工作表现是否良好的适当反馈。 | □ | □ | □ | □ | □ |
| FB2 | 我有很多机会了解自己工作完成得如何。 | □ | □ | □ | □ | □ |
| FB3 | 我的工作成果能够得到适当的认可或回报。 | □ | □ | □ | □ | □ |
| SD1 | 我能通过工作不断学习新的内容。 | □ | □ | □ | □ | □ |
| SD2 | 所在单位为我提供充分的自我发展机会。 | □ | □ | □ | □ | □ |
| SD3 | 所在单位为我提供发挥自身优势的机会。 | □ | □ | □ | □ | □ |

## 第三部分 工作自我效能

| 编号 | 陈述 | 1 | 2 | 3 | 4 | 5 |
|---|---|---|---|---|---|---|
| JSE1 | 我具备完成本职工作所需的能力。 | □ | □ | □ | □ | □ |
| JSE2 | 在自己的工作领域，我认为自己具备专业胜任能力。 | □ | □ | □ | □ | □ |
| JSE3 | 我对自己的工作能力有信心。 | □ | □ | □ | □ | □ |

## 第四部分 工作投入

| 编号 | 陈述 | 1 | 2 | 3 | 4 | 5 |
|---|---|---|---|---|---|---|
| WE1 | 工作时我感到精力充沛。 | □ | □ | □ | □ | □ |
| WE2 | 早晨醒来时，我会产生去工作的意愿。 | □ | □ | □ | □ | □ |
| WE3 | 我对自己的工作充满热情。 | □ | □ | □ | □ | □ |

## 第五部分 工作满意度

| 编号 | 陈述 | 1 | 2 | 3 | 4 | 5 |
|---|---|---|---|---|---|---|
| JS1 | 总体而言，我对目前从事的工作感到满意。 | □ | □ | □ | □ | □ |
| JS2 | 我能从工作中获得成就感。 | □ | □ | □ | □ | □ |
| JS3 | 我从事的工作很有趣。 | □ | □ | □ | □ | □ |
| JS4 | 我的工作与自身能力水平较为匹配。 | □ | □ | □ | □ | □ |
| JS5 | 我把单位的工作视为自己的工作。 | □ | □ | □ | □ | □ |
| JS6 | 我很高兴在当前单位工作。 | □ | □ | □ | □ | □ |

## 第六部分 基本信息

1. 性别： □ 男 □ 女

2. 职业角色： □ 教练员 □ 运动员 □ 裁判/技术保障人员

3. 主要从事/指导项目：________________________________

4. 所在地区： □ 华北 □ 东北 □ 华东 □ 华中 □ 华南 □ 西南 □ 西北
