# Supplementary material for: Contextual boundaries of personal resource translation: dual-path associations of role overload and job autonomy with job satisfaction among sports practitioners
Source: Front Psychol. 2026 Oct 7;17:1844634. doi: 10.3389/fpsyg.2026.1844634 (PMC13682992; doi:10.3389/fpsyg.2026.1844634)
Supplement: Supplementary file 2 [file Data_Sheet_2.ZIP › Supplementary Materials/Questionnaire_English_Translation.pdf]

Supplementary Questionnaire

Survey of Job Demands, Job Resources, and Occupational Psychological States among Sports Practitioners

English Translation

Dear Participant,
Thank you for taking the time to participate in this survey.

This questionnaire is designed to understand the experiences of sports practitioners with respect to job demands, job resources, job self-efficacy, work engagement, and job satisfaction. There are no right or wrong answers. Please respond honestly according to your current work experience.

This survey is for academic research only. Participation is completely voluntary. You may decline to participate or stop completing the questionnaire at any time without any adverse consequence. Questionnaire information will be kept confidential and analyzed in de-identified form and will not be used for purposes outside the research. No financial compensation is provided.

The study was approved by the Ethics Committee of Yancheng Institute of Technology (YIT-TY-IRB-2025-07-007). If you agree to participate, please proceed after completing written informed consent. Thank you for your understanding and support.

Instructions. Except for the demographic questions, all statements use a five-point Likert scale: 1 = strongly disagree, 2 = disagree, 3 = neither agree nor disagree, 4 = agree, and 5 = strongly agree. Select one response for each statement.

Part I. Job Demands

Code	Statement	1	2	3	4	5
RO1	Work needs to be handled quickly.	□	□	□	□	□
RO2	I have too much work to do.	□	□	□	□	□
RO3	I have to work very hard to complete my work.	□	□	□	□	□
RO4	I often work under time pressure.	□	□	□	□	□
RO5	I often have to rush to finish my work.	□	□	□	□	□
RO6	I feel that I cannot keep up with my current workload.	□	□	□	□	□
RA1	I am not clear about what authority I have in my work.	□	□	□	□	□
RA2	My work tasks or goals are not clear.	□	□	□	□	□
RA3	When completing my work, I am not clear about how I should allocate my time.	□	□	□	□	□
RA4	I am not clear about which responsibilities I should assume at work.	□	□	□	□	□
RA5	I am not clear about what my organization expects of me.	□	□	□	□	□
RA6	When carrying out my work, I do not receive clear instructions about work methods.	□	□	□	□	□
RC1	I lack the information needed to complete my work.	□	□	□	□	□
RC2	To complete my work, I sometimes have to violate the rules or policies of my organization.	□	□	□	□	□
RC3	I am sometimes asked by different people to perform different types of work.	□	□	□	□	□
RC4	I sometimes undertake tasks unrelated to my primary duties.	□	□	□	□	□
RC5	I sometimes undertake work that does not receive recognition or support from others.	□	□	□	□	□

Part II. Job Resources

Code	Statement	1	2	3	4	5
SS1	My colleagues care about my emotions and feelings.	□	□	□	□	□
SS2	My colleagues care about the problems I encounter.	□	□	□	□	□
SS3	My colleagues sincerely help me with work-related problems.	□	□	□	□	□
SS4	I work in a good working atmosphere.	□	□	□	□	□
JA1	I have independence in how I carry out my work.	□	□	□	□	□
JA2	I have considerable freedom in how I carry out my work.	□	□	□	□	□
JA3	I can negotiate with colleagues to adjust my working time.	□	□	□	□	□
JA4	At work, I am not excessively constrained by cumbersome procedures.	□	□	□	□	□
FB1	I receive appropriate feedback about whether I am performing my work well.	□	□	□	□	□
FB2	I have many opportunities to find out how well I have completed my work.	□	□	□	□	□
FB3	My work results receive appropriate recognition or reward.	□	□	□	□	□

Code	Statement	1	2	3	4	5
SD1	I can continue to learn new things through my work.	□	□	□	□	□
SD2	My organization provides sufficient opportunities for my self-development.	□	□	□	□	□
SD3	My organization provides opportunities for me to use my strengths.	□	□	□	□	□

Part III. Job Self-Efficacy

Code	Statement	1	2	3	4	5
JSE1	I have the abilities needed to perform my job.	□	□	□	□	□
JSE2	In my area of work, I consider myself professionally competent.	□	□	□	□	□
JSE3	I am confident in my work abilities.	□	□	□	□	□

Part IV. Work Engagement

Code	Statement	1	2	3	4	5
WE1	I feel energetic while working.	□	□	□	□	□
WE2	When I wake up in the morning, I feel willing to go to work.	□	□	□	□	□
WE3	I am enthusiastic about my work.	□	□	□	□	□

Part V. Job Satisfaction

Code	Statement	1	2	3	4	5
JS1	Overall, I am satisfied with my current work.	□	□	□	□	□
JS2	I gain a sense of accomplishment from my work.	□	□	□	□	□
JS3	The work I do is interesting.	□	□	□	□	□
JS4	My work is well matched to my level of ability.	□	□	□	□	□
JS5	I regard the work of my organization as my own work.	□	□	□	□	□
JS6	I am happy to work in my current organization.	□	□	□	□	□

Part VI. Demographic Information

1. Sex: □ Male □ Female

2. Occupational role: □ Coach □ Athlete □ Referee / technical-support personnel

3. Primary sport in which you work / provide instruction: ____

4. Region: □ North China □ Northeast China □ East China □ Central China □ South China □ Southwest China □ Northwest China

Note. This English document is a translation provided for reference. The Chinese questionnaire was the version administered to participants.
