# Supplementary material for: Contextual boundaries of personal resource translation: dual-path associations of role overload and job autonomy with job satisfaction among sports practitioners
Source: Front Psychol. 2026 Oct 7;17:1844634. doi: 10.3389/fpsyg.2026.1844634 (PMC13682992; doi:10.3389/fpsyg.2026.1844634)
Supplement: Supplementary file 2 [file Data_Sheet_2.ZIP › Supplementary Materials/Supplementary_Information.pdf]

Supplementary Information

This supplement provides additional information on recruitment and sample characteristics, the broader 43-item questionnaire pool, the chronology and rationale for the five focal constructs, item-level measurement results, common-method sensitivity analyses, conditional-process coefficients, diagnostics, measurement invariance, and occupational-role sensitivity analyses.

Table S0. Recruitment, questionnaire flow, eligibility, and available demographic information

Item	Verifiable information
Data-collection period	1 August-30 September 2025
Mode	Entirely offline, paper-and-pencil questionnaires
Recruitment channels	Sports bureaus / sports teams / training bases; sports associations / clubs; acquaintance referral / snowball sampling
Recruitment frame	Adults engaged in sport-related roles
Adult-status verification	Research-team members inspected each participant's government-issued identity document before questionnaire administration. No identity-document number or other identifying information was recorded.
Written consent	Written informed consent / confirmation was obtained before participation
Complete questionnaires returned	358
Excluded after receipt	0
Final analytic sample	358
Conventional response rate	Not calculated because the total number of paper questionnaires initially distributed across multiple recruitment channels was not retained in one unified, verifiable record
Demographic fields recorded on questionnaire	Sex; occupational role; primary sport; broad region
Row-level demographic fields available in the analytic file	Sex; occupational role; anonymous six-level archived category code
Demographic variables not collected	Age as a numeric variable; education; employment status; occupational tenure
Primary sport / region linkage	Only aggregate manual paper tallies remain for primary sport and region; they cannot be linked row-by-row to focal outcomes and are not included in participant-level descriptive tables
Compensation / gifts	None
Ethics	YIT-TY-IRB-2025-07-007; approved 30 June 2025

Table S1A. Construct coverage in the broader questionnaire and focal analysis

Construct	Items in broader survey	Analytic role
Role overload	6	Included in focal analysis; interpreted narrowly as quantitative workload/time-pressure overload
Role ambiguity	6	Administered in broader survey; not included in this focal article
Role conflict	5	Administered in broader survey; not included in this focal article
Social support	4	Administered in broader survey; not included in this focal article
Job autonomy	4	Included in focal analysis
Self-development opportunities	3	Administered in broader survey; not included in this focal article
Feedback	3	Administered in broader survey; not included in this focal article
Job self-efficacy	3	Included in focal analysis
Work engagement	3	Included in focal analysis
Job satisfaction	6	Included in focal analysis; contextualized favorable work-evaluation composite

Note. The broader survey contained 43 non-demographic measurement items spanning multiple job demands, job resources, and work-related psychological variables. The focal analysis uses 22 items representing five constructs.

Table S1B. Focal-framework specification and construct-selection rationale

Issue	Description
Timing of focal-framework specification	The focal framework was specified after completion of data collection.
Empirical information available before focal-framework specification	Descriptive statistics/correlations; CFA/reliability/validity results; ordinary regression and Model 4 indirect-association results.
Conditional analyses conducted after focal-framework specification	Model 7 interaction results; Johnson-Neyman regions; moderated-mediation indices.
Analytic status	Post hoc, theory-guided analysis informed by preliminary descriptive/correlational, measurement-model, and ordinary regression/Model 4 results.
Theoretical rationale for the focal constructs	Role overload provided a quantitative demand-intensity indicator; job autonomy provided an action-control indicator; job self-efficacy represented a work-domain capability belief; work engagement represented a proximal occupational state; job satisfaction represented the focal global work-evaluation outcome.

Issue	Description
Scope of the other administered constructs	Role ambiguity, role conflict, social support, feedback, and self-development opportunities address distinct questions concerning role clarity/conflict and alternative job resources and fall outside the focal dual-path question examined here.
Item retention within focal constructs	The focal scoring sets comprise all six RO, four JA, three JSE, three WE, and six JS items.

Table S1C. Focal 22 items, scoring, and source lineage

Variable	Construct	English translation of administered Chinese item	Scoring	Source lineage / interpretation
RO1	Role overload (quantitative workload/time-pressure facet)	Work needs to be handled quickly.	1–5; higher = more role overload; no reverse scoring	Contextualized quantitative-overload item; content overlaps established role-overload formulations centered on excessive work/insufficient time and quantitative workload traditions (Bolino & Turnley, 2005; Spector & Jex, 1998).
RO2	Role overload (quantitative workload/time-pressure facet)	I have too much work to do.	1–5; higher = more role overload; no reverse scoring	Same quantitative-overload source lineage as RO1.
RO3	Role overload (quantitative workload/time-pressure facet)	I have to work very hard to complete my work.	1–5; higher = more role overload; no reverse scoring	Same quantitative-overload source lineage as RO1.
RO4	Role overload (quantitative workload/time-pressure facet)	I often work under time pressure.	1–5; higher = more role overload; no reverse scoring	Same quantitative-overload source lineage as RO1.
RO5	Role overload (quantitative workload/time-pressure facet)	I often have to rush to finish my work.	1–5; higher = more role overload; no reverse scoring	Same quantitative-overload source lineage as RO1.
RO6	Role overload (quantitative workload/time-pressure facet)	I feel that I cannot keep up with my current workload.	1–5; higher = more role overload; no reverse scoring	Same quantitative-overload source lineage as RO1.
JA1	Job autonomy	I have independence in how I carry out my work.	1–5; higher = more job autonomy; no reverse scoring	Contextualized job-autonomy item; conceptually aligned with Morgeson & Humphrey (2006)
JA2	Job autonomy	I have considerable freedom in how I carry out my work.	1–5; higher = more job autonomy; no reverse scoring	Same source lineage as JA1
JA3	Job autonomy	I can negotiate with colleagues to adjust my working time.	1–5; higher = more job autonomy; no reverse scoring	Same source lineage as JA1
JA4	Job autonomy	At work, I am not excessively constrained by cumbersome procedures.	1–5; higher = more job autonomy; no reverse scoring	Same source lineage as JA1
JSE1	Job self-efficacy	I have the abilities needed to perform my job.	1–5; higher = greater job self-efficacy; no reverse scoring	Job-related self-efficacy lineage; Riggs et al. (1994), contextualized to sport work
JSE2	Job self-efficacy	In my area of work, I consider myself professionally competent.	1–5; higher = greater job self-efficacy; no reverse scoring	Same source lineage as JSE1
JSE3	Job self-efficacy	I am confident in my work abilities.	1–5; higher = greater job self-efficacy; no reverse scoring	Same source lineage as JSE1
WE1	Work engagement	I feel energetic while working.	1–5; higher = greater work engagement; no reverse scoring	Contextualized short work-engagement content following the UWES short-form tradition; primarily representing vigor and enthusiasm/dedication.
WE2	Work engagement	When I wake up in the morning, I feel willing to go to work.	1–5; higher = greater work engagement; no reverse scoring	Same source lineage as WE1
WE3	Work engagement	I am enthusiastic about my work.	1–5; higher = greater work engagement; no reverse scoring	Same source lineage as WE1
JS1	Job satisfaction	Overall, I am satisfied with my current work.	1–5; higher = greater job satisfaction; no reverse scoring	Contextualized job-satisfaction / favorable work-evaluation item set.
JS2	Job satisfaction	I gain a sense of accomplishment from my work.	1–5; higher = greater job satisfaction; no reverse scoring	Same item-set lineage as JS1
JS3	Job satisfaction	The work I do is interesting.	1–5; higher = greater job satisfaction; no reverse scoring	Same item-set lineage as JS1
JS4	Job satisfaction	My work is well matched to my level of ability.	1–5; higher = greater job satisfaction; no reverse scoring	Same item-set lineage as JS1
JS5	Job satisfaction	I regard the work of my organization as my own work.	1–5; higher = greater job satisfaction; no reverse scoring	Same item-set lineage as JS1
JS6	Job satisfaction	I am happy to work in my current organization.	1–5; higher = greater job satisfaction; no reverse scoring	Same item-set lineage as JS1

Note. The Chinese wording in the administered questionnaire is provided in the separate clean blank Supplementary Questionnaire. The English wording is a translation provided for reference and was not administered to participants. All 22 focal items were scored in the same direction; no reverse scoring was used.

Table S1D. Item-level standardized loadings in the five-factor CFA

Item	Construct	Standardized loading
RO1	Role overload	0.716

Item	Construct	Standardized loading
RO2	Role overload	0.700
RO3	Role overload	0.872
RO4	Role overload	0.712
RO5	Role overload	0.752
RO6	Role overload	0.706
JA1	Job autonomy	0.760
JA2	Job autonomy	0.769
JA3	Job autonomy	0.812
JA4	Job autonomy	0.725
JSE1	Job self-efficacy	0.724
JSE2	Job self-efficacy	0.734
JSE3	Job self-efficacy	0.816
WE1	Work engagement	0.762
WE2	Work engagement	0.772
WE3	Work engagement	0.758
JS1	Job satisfaction	0.747
JS2	Job satisfaction	0.767
JS3	Job satisfaction	0.713
JS4	Job satisfaction	0.837
JS5	Job satisfaction	0.734
JS6	Job satisfaction	0.778

Note. These are the item-level standardized loadings underlying the ranges reported in main-text Table 2. All loadings are from the five-factor ML solution (chi-square = 382.939, df = 199).

Table S2A. Common-method sensitivity model fit

Model	chi-square	df	chi-square/df	RMSEA	CFI	TLI	SRMR
Five-factor CFA	382.939	199	1.924	0.051	0.952	0.944	0.058
One-factor CFA	2035.089	209	9.737	0.156	0.525	0.475	0.140
Equal-loading general method factor	382.939	198	1.934	0.051	0.952	0.944	0.058
Unrestricted general-factor sensitivity model	215.352	177	1.217	0.025	0.990	0.987	0.029

Note. No independent marker variable was included in the questionnaire. Accordingly, the unrestricted orthogonal general-factor model is used as a sensitivity analysis of shared evaluative covariance rather than as a direct estimate of common-method variance.

Table S2B. Unrestricted general-factor loading pattern and solution diagnostics

Item	Construct	Substantive std. loading	General-factor std. loading	Std. residual variance
RO1	Role overload	0.577	−0.418	0.492
RO2	Role overload	0.541	−0.458	0.498
RO3	Role overload	0.895	−0.261	0.131
RO4	Role overload	0.585	−0.384	0.511
RO5	Role overload	0.641	−0.388	0.438
RO6	Role overload	0.595	−0.364	0.514
JA1	Job autonomy	0.510	0.567	0.419
JA2	Job autonomy	0.527	0.553	0.416
JA3	Job autonomy	0.801	0.371	0.221
JA4	Job autonomy	0.570	0.433	0.487
JSE1	Job self-efficacy	0.350	0.602	0.515
JSE2	Job self-efficacy	0.390	0.586	0.504
JSE3	Job self-efficacy	0.782	0.542	0.095
WE1	Work engagement	0.799	0.362	0.231
WE2	Work engagement	0.505	0.555	0.438

Item	Construct	Substantive std. loading	General-factor std. loading	Std. residual variance
WE3	Work engagement	0.513	0.532	0.454
JS1	Job satisfaction	0.479	0.565	0.451
JS2	Job satisfaction	0.509	0.568	0.419
JS3	Job satisfaction	0.455	0.545	0.496
JS4	Job satisfaction	0.690	0.533	0.239
JS5	Job satisfaction	0.484	0.551	0.463
JS6	Job satisfaction	0.462	0.622	0.399

Note. In the unrestricted orthogonal general-factor sensitivity model, all six role-overload items loaded negatively on the general factor (−0.261 to −0.458), whereas the 16 job-autonomy, job-self-efficacy, work-engagement, and job-satisfaction items loaded positively (0.362 to 0.622), a pattern consistent with evaluative polarity. Standardized residual variances were all positive (0.095–0.515), no absolute standardized loading exceeded 1, and no Heywood-type improper estimate was evident. Model fit was chi-square = 215.352, df = 177, RMSEA = 0.025, CFI = 0.990, TLI = 0.987, SRMR = 0.029.

Table S3. Full coefficients for primary Model 7 and joint models (HC3)

Equation	Variable	B	HC3 SE	z	p	95% CI	R^2
RO Model 7: WE equation	Intercept	3.385	0.066	51.337	< .001	[3.256, 3.514]	0.224
RO Model 7: WE equation	ROc	−0.094	0.057	-1.656	.098	[−0.205, 0.017]	0.224
RO Model 7: WE equation	JSEc	0.455	0.052	8.770	< .001	[0.353, 0.556]	0.224
RO Model 7: WE equation	RO × JSE	−0.152	0.057	-2.660	.008	[−0.265, −0.040]	0.224
RO Model 7: WE equation	Female	−0.010	0.100	−0.095	.924	[−0.205, 0.186]	0.224
RO Model 7: JS equation	Intercept	1.786	0.165	10.846	< .001	[1.463, 2.109]	0.300
RO Model 7: JS equation	ROc	−0.265	0.050	-5.346	< .001	[−0.363, −0.168]	0.300
RO Model 7: JS equation	WE	0.415	0.043	9.598	< .001	[0.330, 0.500]	0.300
RO Model 7: JS equation	Female	0.144	0.087	1.642	.101	[−0.028, 0.315]	0.300
JA Model 7: WE equation	Intercept	3.399	0.067	50.929	< .001	[3.268, 3.530]	0.218
JA Model 7: WE equation	JAc	0.113	0.058	1.945	.052	[−0.001, 0.226]	0.218
JA Model 7: WE equation	JSEc	0.442	0.056	7.864	< .001	[0.332, 0.553]	0.218
JA Model 7: WE equation	JA × JSE	0.106	0.057	1.860	.063	[−0.006, 0.218]	0.218
JA Model 7: WE equation	Female	−0.034	0.101	−0.338	.736	[−0.231, 0.163]	0.218
JA Model 7: JS equation	Intercept	1.777	0.166	10.729	< .001	[1.452, 2.101]	0.277
JA Model 7: JS equation	JAc	0.193	0.048	4.017	< .001	[0.099, 0.287]	0.277
JA Model 7: JS equation	WE	0.418	0.043	9.640	< .001	[0.333, 0.502]	0.277
JA Model 7: JS equation	Female	0.144	0.089	1.620	.105	[−0.030, 0.318]	0.277
Joint Model: WE equation	Intercept	3.377	0.069	49.249	< .001	[3.243, 3.511]	0.231
Joint Model: WE equation	ROc	−0.058	0.058	−0.989	.323	[−0.172, 0.057]	0.231
Joint Model: WE equation	JAc	0.070	0.060	1.160	.246	[−0.048, 0.187]	0.231
Joint Model: WE equation	JSEc	0.446	0.057	7.882	< .001	[0.335, 0.557]	0.231
Joint Model: WE equation	RO × JSE	−0.124	0.060	-2.078	.038	[−0.242, −0.007]	0.231
Joint Model: WE equation	JA × JSE	0.073	0.061	1.206	.228	[−0.046, 0.192]	0.231
Joint Model: WE equation	Female	−0.023	0.101	−0.229	.819	[−0.220, 0.174]	0.231
Joint Model: JS equation	Intercept	1.869	0.170	10.969	< .001	[1.535, 2.203]	0.319
Joint Model: JS equation	ROc	−0.229	0.052	-4.406	< .001	[−0.331, −0.127]	0.319
Joint Model: JS equation	JAc	0.143	0.051	2.786	.005	[0.042, 0.244]	0.319
Joint Model: JS equation	WE	0.391	0.045	8.655	< .001	[0.302, 0.479]	0.319
Joint Model: JS equation	Female	0.144	0.087	1.666	.096	[−0.025, 0.314]	0.319

Note. ROc, JAc, and JSEc are sample-mean-centered variables. Female = 1; male is the reference category. B values are unstandardized coefficients. Numerical p values are shown to three decimals when p >= .001; smaller values are reported as p < .001. Primary models adjust only for sex.

Table S4. Conditional indirect associations and bootstrap results

Path	JSE level / index	Effect	Boot SE	95% Boot CI
RO	Low JSE (−1 SD)	0.023	0.038	[−0.053, 0.097]
RO	Mean JSE	−0.039	0.023	[−0.087, 0.006]
RO	High JSE (+1 SD)	−0.101	0.028	[−0.161, −0.051]

Path	JSE level / index	Effect	Boot SE	95% Boot CI
RO	Index of moderated mediation	−0.063	0.024	[−0.111, −0.016]
JA	Low JSE (−1 SD)	0.003	0.037	[−0.071, 0.075]
JA	Mean JSE	0.047	0.023	[0.002, 0.093]
JA	High JSE (+1 SD)	0.091	0.029	[0.036, 0.152]
JA	Index of moderated mediation	0.044	0.024	[−0.001, 0.093]
Joint RO	Indirect effect	−0.062	0.025	[−0.114, −0.014]
Joint JA	Indirect effect	0.078	0.024	[0.033, 0.128]
Joint RO	Index of moderated mediation	−0.049	0.024	[−0.096, −0.003]
Joint JA	Index of moderated mediation	0.029	0.023	[−0.016, 0.075]

Note. Nonparametric bootstrap = 10,000 resamples; random seed = 20260912.

Table S5. Diagnostics, Johnson-Neyman threshold, and anonymous sport-code sensitivity

Diagnostic	Result
Maximum VIF	1.233
Maximum Cook's distance	0.035
Maximum |externally studentized residual|	2.725
Breusch–Pagan LM	31.658
Breusch–Pagan p	< .001

Johnson-Neyman threshold: JSE = 3.364. On the original 1-5 JSE scale, the adjusted RO -> WE slope is significantly negative above this threshold (two-sided alpha = .05).

The electronic analysis archive contains a valid six-level categorical code whose semantic labels cannot be recovered reliably. It is therefore treated only as an anonymous sensitivity covariate and is not given a substantive sport-type interpretation. Primary sport and broad region are available only as aggregate manual paper tallies and are not included in participant-level descriptive analyses because no row-level linkage to the analytic records is available.

Sensitivity model	Interaction	B	HC3 SE	p	95% CI
RO Model 7 + sport code	RO × JSE	−0.152	0.058	.009	[−0.266, −0.038]
JA Model 7 + sport code	JA × JSE	0.109	0.058	.059	[−0.004, 0.221]
Joint + sport code	RO × JSE	−0.121	0.061	.047	[−0.240, −0.002]
Joint + sport code	JA × JSE	0.077	0.061	.205	[−0.042, 0.195]

Table S6. Reproducibility materials and variable coding

Item	Content
Primary covariate	Sex; 1 = male, 2 = female; male is the reference in regression models
Anonymous sport code	1–6; valid row-level categorical code; semantic labels not recoverable; sensitivity only
Focal items	22 items, all scored 1–5 in the same direction; no reverse scoring
Construct scoring	Mean of constituent items
Centering	RO, JA, and JSE were sample-mean centered before interaction terms were created
Bootstrap	10,000 resamples; seed = 20260912
Reproducibility software	R/RStudio analysis environment; scripts write sessionInfo() when run
Data-sharing mode	Not posted publicly; de-identified data, codebook, and R scripts available on reasonable request
Occupational role	Row-level field: coach n=128, athlete n=162, referee/technical support n=68; used for three-group MG-CFA and role-adjusted sensitivity analyses
Age / education / employment / tenure	Age was not recorded as a numeric analytic variable; education, employment status, and occupational tenure were not collected. Adult eligibility was verified on site from identity documents without recording identifiers.
Primary sport / region	Collected on paper questionnaires, but only aggregate manual tallies remain; no row-level linkage to outcomes is available.
Measurement outputs	item_level_five_factor_loadings.csv; unrestricted_general_factor_loadings.csv; unrestricted_general_factor_diagnostics.csv

Table S7. Multi-group measurement invariance across occupational roles

Model	χ^2	df	χ^2/df	CFI	TLI	RMSEA	SRMR	ΔCFI	ΔRMSEA
Configural	835.968	597	1.400	0.938	0.928	0.034	0.073		

Model	χ²	df	χ²/df	CFI	TLI	RMSEA	SRMR	ΔCFI	ΔRMSEA
Metric	873.262	631	1.384	0.938	0.931	0.033	0.079	−0.001	−0.001
Scalar	913.713	665	1.374	0.936	0.933	0.032	0.079	−0.002	−0.001
Strict	940.064	709	1.326	0.940	0.942	0.030	0.080	+0.005	−0.002

Note. Groups: coaches (n = 128), athletes (n = 162), and referees/technical-support personnel (n = 68). Metric vs configural: delta chi-square = 37.294, delta df = 34, p = .320; scalar vs metric: delta chi-square = 40.451, delta df = 34, p = .207; strict vs scalar: delta chi-square = 26.351, delta df = 44, p = .984. Changes in CFI and RMSEA are within commonly used invariance criteria.

Table S8. Conditional-process sensitivity analyses adjusted for occupational role

Model	Statistic	B / effect	HC3 SE / Boot SE	p	95% CI	Inference
RO Model 7 + role	RO × JSE	−0.151	0.058	.009	[−0.264, −0.037]	Supported
JA Model 7 + role	JA × JSE	0.105	0.057	.064	[−0.006, 0.217]	Not supported
Joint + role	RO × JSE	−0.123	0.060	.042	[−0.241, −0.005]	Supported
Joint + role	JA × JSE	0.071	0.060	.242	[−0.048, 0.189]	Not supported
RO Model 7 + role	Index of moderated mediation	−0.062	0.024		[−0.110, −0.015]	Supported
JA Model 7 + role	Index of moderated mediation	0.044	0.024		[−0.001, 0.092]	Not supported
Joint + role	RO index of moderated mediation	−0.048	0.024		[−0.095, −0.002]	Supported
Joint + role	JA index of moderated mediation	0.027	0.023		[−0.016, 0.074]	Not supported

Note. Coach is the reference category; dummy variables for athlete and referee/technical-support personnel were added, with sex also adjusted. Bootstrap = 10,000 resamples; seed = 20260912. The direction and inferential classification of the focal RO x JSE pattern and its moderated indirect association were unchanged after occupational-role adjustment.
